# Supplementary material for: Atomic-resolution structure analysis inside an adaptable porous framework
Source: Nat Commun. 2024 Jan 2;15:81. doi: 10.1038/s41467-023-44401-w (PMC10762011; doi:10.1038/s41467-023-44401-w)
Supplement: Supplementary file 1 — Supplementary Information [file 41467_2023_44401_MOESM1_ESM.pdf]

Supplementary Information

Atomic-resolution structure analysis inside an adaptable porous framework

Yuki Wada[1], Pavel M Usov[1], Bun Chan[2], Makoto Mukaida[3], Ken Ohmori[1], Yoshio Ando[1], Haruhiko Fuwa[4], Hiroyoshi Ohtsu[1] and Masaki Kawano[1]*.

Corresponding author: mkawano@chem.titech.ac.jp

Supplementary Methods
Supplementary Fig 1 to 18
Supplementary Tab 1 to 5
Supplementary References

Supplementary methods

Synthesis of K(3-TPHAP)

The K(3-TPHAP) was synthesized according to the published procedure.[1]

3-amidinopyridine hydrochloride (4.00 g, 26.0 mmol) and potassium tricyanomethanide (0.86 g, 6.7 mmol) were placed in a Teflon-lined stainless-steel autoclave and heated at 180 °C for 20 h. After heating, the resultant black solid was ground and dissolved in water (300 mL). The solution was stirred for 5 h, after which it was filtered, and the filtrate washed with ethyl acetate (*ca.* 100 mL) 5 times. The aqueous layer was reduced to dryness, followed by addition of 5 M aqueous HCl solution (*ca.* 20 mL). Addition of acetone to the resultant solution caused precipitation of a brown-yellow solid, which was isolated by vacuum filtration. The solid was re-dissolved in water, and the solution was neutralized using 5 M aqueous KOH solution. The precipitated solid was isolated by vacuum filtration and re-dissolved in methanol (100 mL). Addition of excess amount of acetone caused precipitation of inorganic salts, which were separated. The remaining solution was reduced to dryness, re-dissolved in acetone (200 mL) followed by addition of chloroform (300 mL). After several days, the pure K(3-TPHAP) appeared as a brown-yellow powder, which was collected by vacuum filtration and dried (286 mg, 9 %). ^{1}H NMR (DMSO-d_6): δ = 7.55 (ddd, 3H, J = 0.8, 4.8, 8.0 Hz), 8.69 (dd, 3H, J = 2.0, 8.6 Hz), 8.78 (dt, J = 2.0, 8.4 Hz), 9.61 (d, 3H, J = 2.8 Hz).^{13}C NMR (DMSO-d_6): δ = 103.0, 123.7, 134.6, 135.9, 149.7, 151.7, 166.4, 166.7. Elemental analysis calcd. (%) for $C_{22.7}H_{23.08}KN_9O_{4.84}$ (= K $(C_{22}H_{12}N_9)\cdot(CH_3OH)_{0.70}\cdot(H_2O)_{4.14}$): C, 50.63; H, 4.32; N, 23.41. Found: C, 50.63; H, 4.09; N, 23.41.

Synthesis of Co-3TPHAP

The coordination network, Co-3TPHAP, was synthesized using modification of the reported procedure[1] to obtain better quality single crystals.

Anhydrous $CoBr_2$ (25.1 mg, 107.7 μmol), K(3-TPHAP) (15.8 mg, 35.82 μmol), and 1,4-benzenedicarboxylic acid (17.9 mg, 107.7 μmol) were dissolved in *N,N′*-dimethylformamide (DMF) (10 mL). The reaction mixture was placed in a Teflon-lined stainless-steel autoclave and heated in an oven at 80 °C for 4 days. After cooling to room temperature, the resultant red-purple crystals mixed with suspended gray solid were transferred to a glass vial. The suspension was decanted, leaving the crystals behind. Fresh DMF (10 mL) was added to the vial, and the solution was decanted again. The process was repeated three times. After that, the solvent was exchanged to ethyl acetate using the same decantation procedure. The crystals were stored in ethyl acetate for further solvent exchange and guest encapsulation studies, whereas for the elemental analysis, they were collected by filtration and dried (45 mg, 41%). Elemental analysis calcd (%) for $Co_4\ C_{116.12}\ H_{179.64}\ N_{34.04}\ O_{43.72}$ [$=Co_4(C_{22}H_{12}N_9)_2(C_8H_4O_4)_3(C_3H_7NO)_4\cdot(C_3H_7NO)_{12.04}\cdot(H_2O)_{15.68}$]: C, 46.84; H, 6.06; N, 15.96. Found: C, 46.84; H, 6.06; N, 15.96.

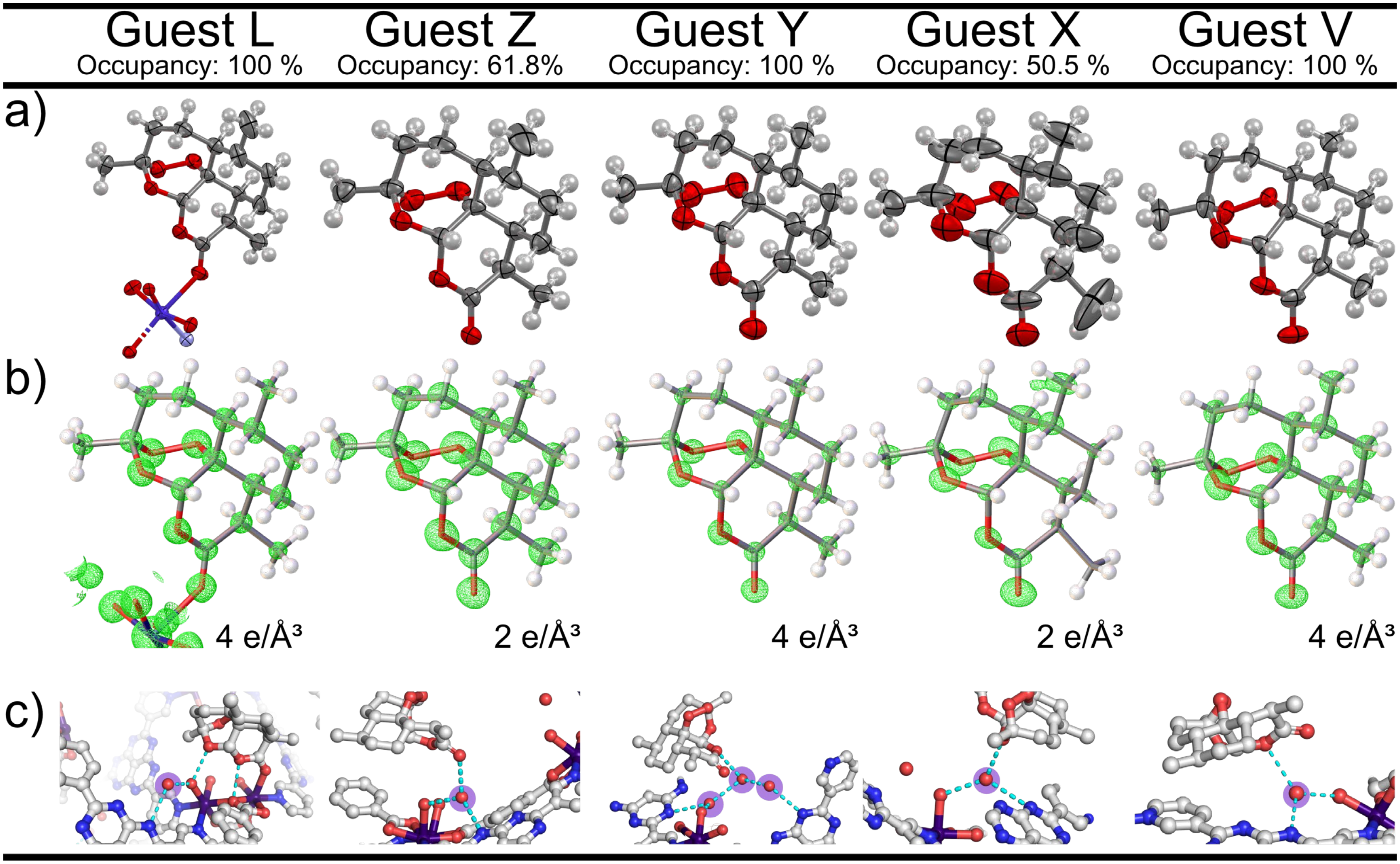

Supplementary Fig 1.

Artemisinin encapsulation summary. a) ORTEP figure with 50% probability, b) 2Fo-Fc electron density map with the number showing the map threshold, c) hydrogen bonding interactions between Co-3TPHAP, water and artemisinin. The water molecules highlighted in purple were freely residing inside the pore, cyan dash lines represent hydrogen bond contacts. Atom coloring scheme: a, b) C: grey, N: blue, O: red, Co: purple, and hydrogen: white; c) C: white, N: blue, O: red, and Co: purple, hydrogen atoms were omitted for clarity.

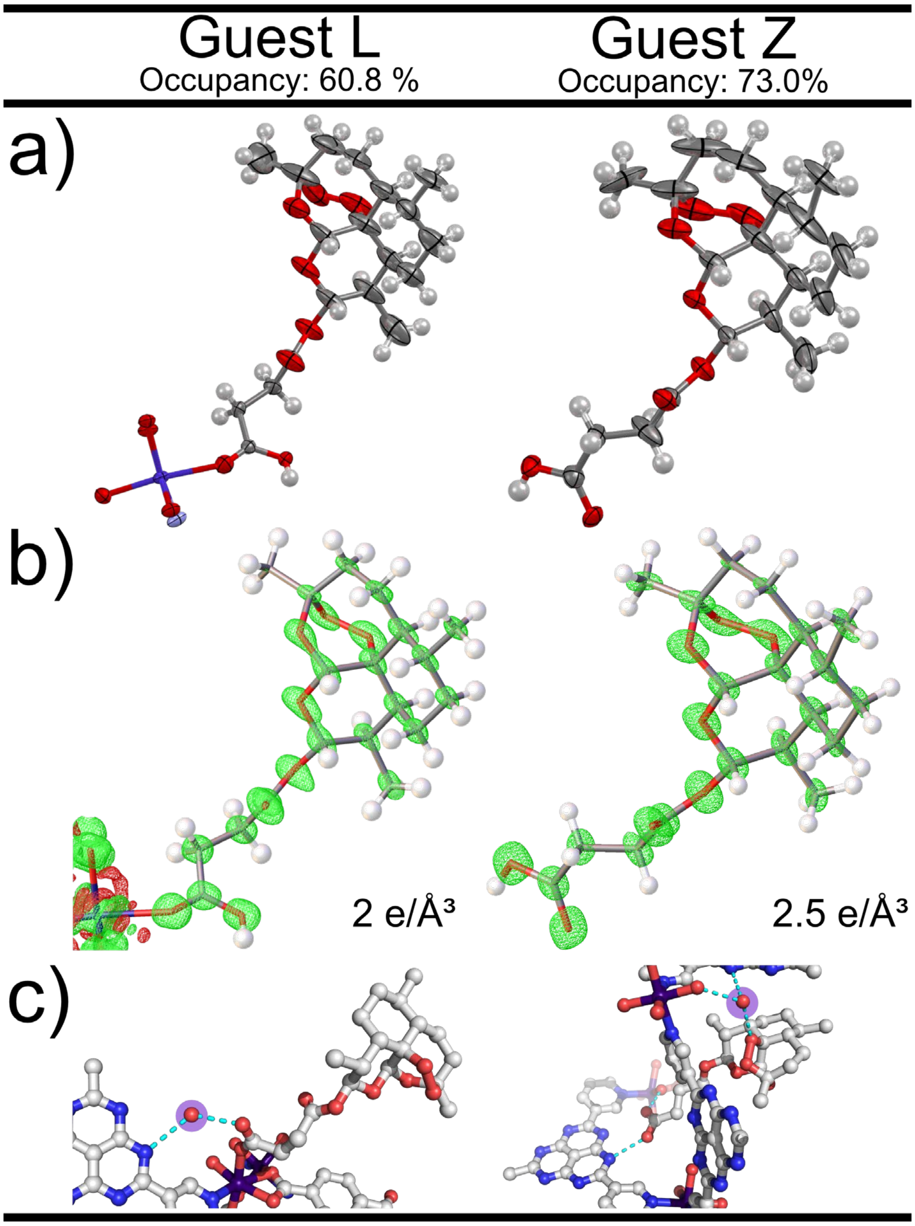

Supplementary Fig 2.
Artesunate encapsulation summary. a) ORTEP figure with 50% probability, b) 2Fo-Fc electron density map with the number showing the map threshold, c) hydrogen bonding interactions between Co-3TPHAP, water and artesunate. The water molecules highlighted in purple were freely residing inside the pore, cyan dash lines represent hydrogen bond contacts. Atom coloring scheme: a, b) C: grey, N: blue, O: red, Co: purple, and hydrogen: white; c) C: white, N: blue, O: red, and Co: purple, hydrogen atoms were omitted for clarity.

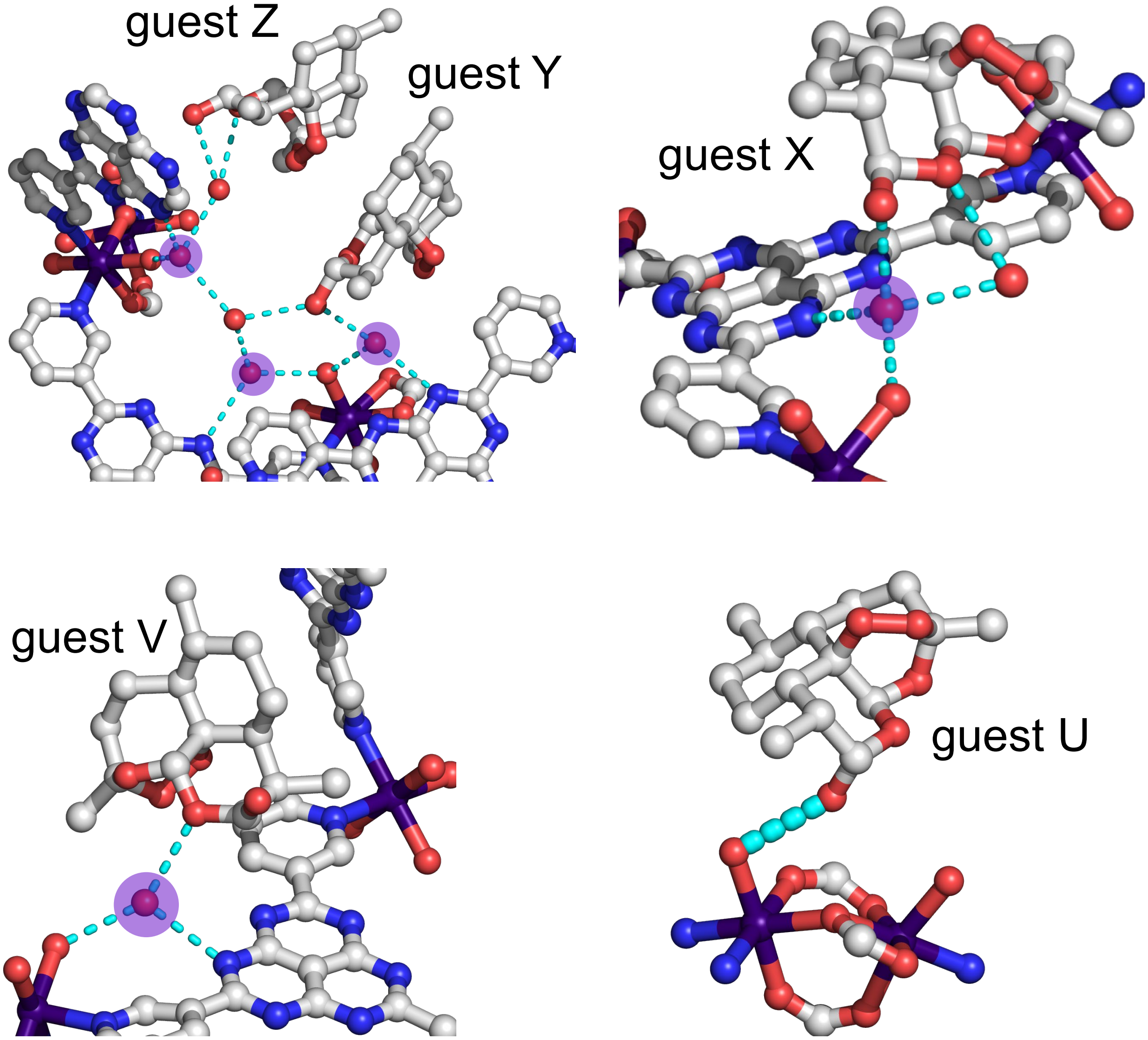

Supplementary Fig 3.
Hydrogen bonding interactions between Co-3TPHAP, water and dihydroartemisinin. The water molecules highlighted in purple were freely residing inside the pore, cyan dash lines represent hydrogen bond contacts. The guest letter corresponds to the suffix in the analysis model. Atom coloring scheme: C: white, N: blue, O: red, and Co: purple, hydrogen atoms were omitted for clarity.

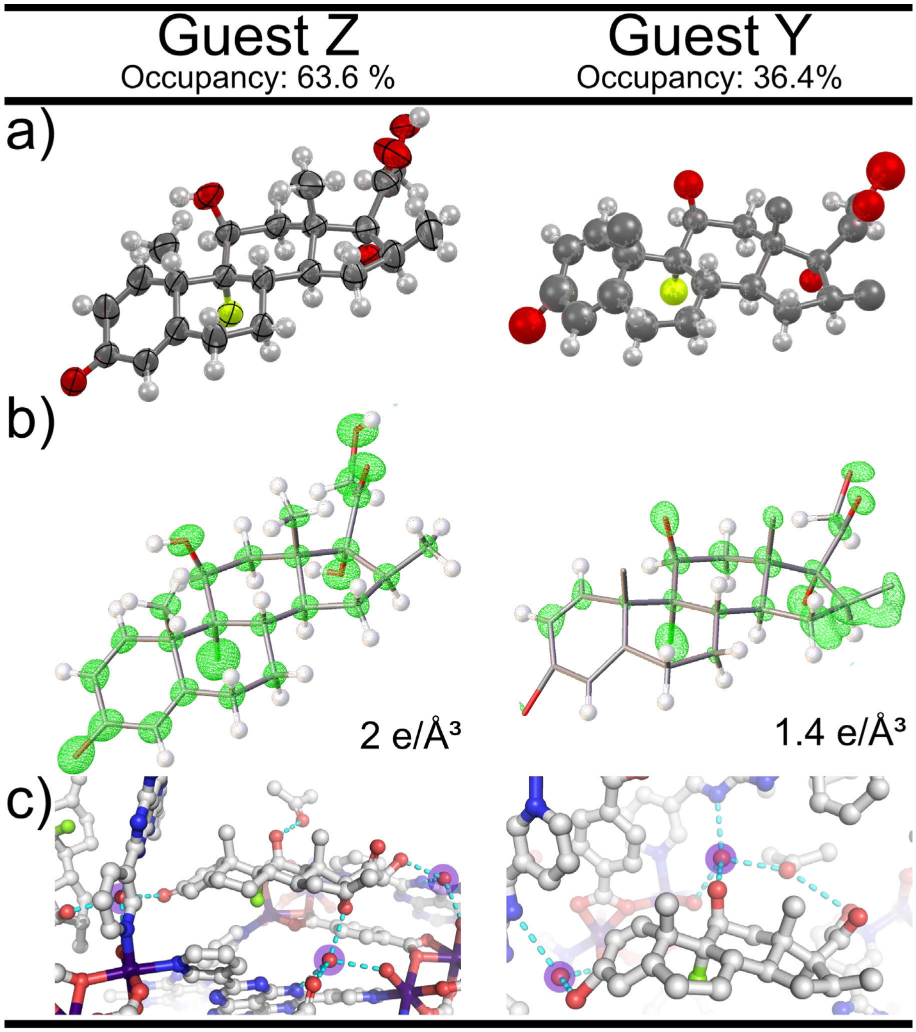

Supplementary Fig 4.
Betamethasone encapsulation summary. a) isotropic and anisotropic models (ORTEP figure) with 50% probability, b) 2Fo-Fc electron density map with the number showing the map threshold, c) hydrogen bonding interactions between Co-3TPHAP, water and betamethasone. The water molecules highlighted in purple were freely residing inside the pore, cyan dash lines represent hydrogen bond contacts. Atom coloring scheme: a, b) C: grey, O: red, F: yellow-green, and hydrogen: white; c) C: white, N: blue, O: red, F: yellow-green and Co: purple, hydrogen atoms were omitted for clarity.

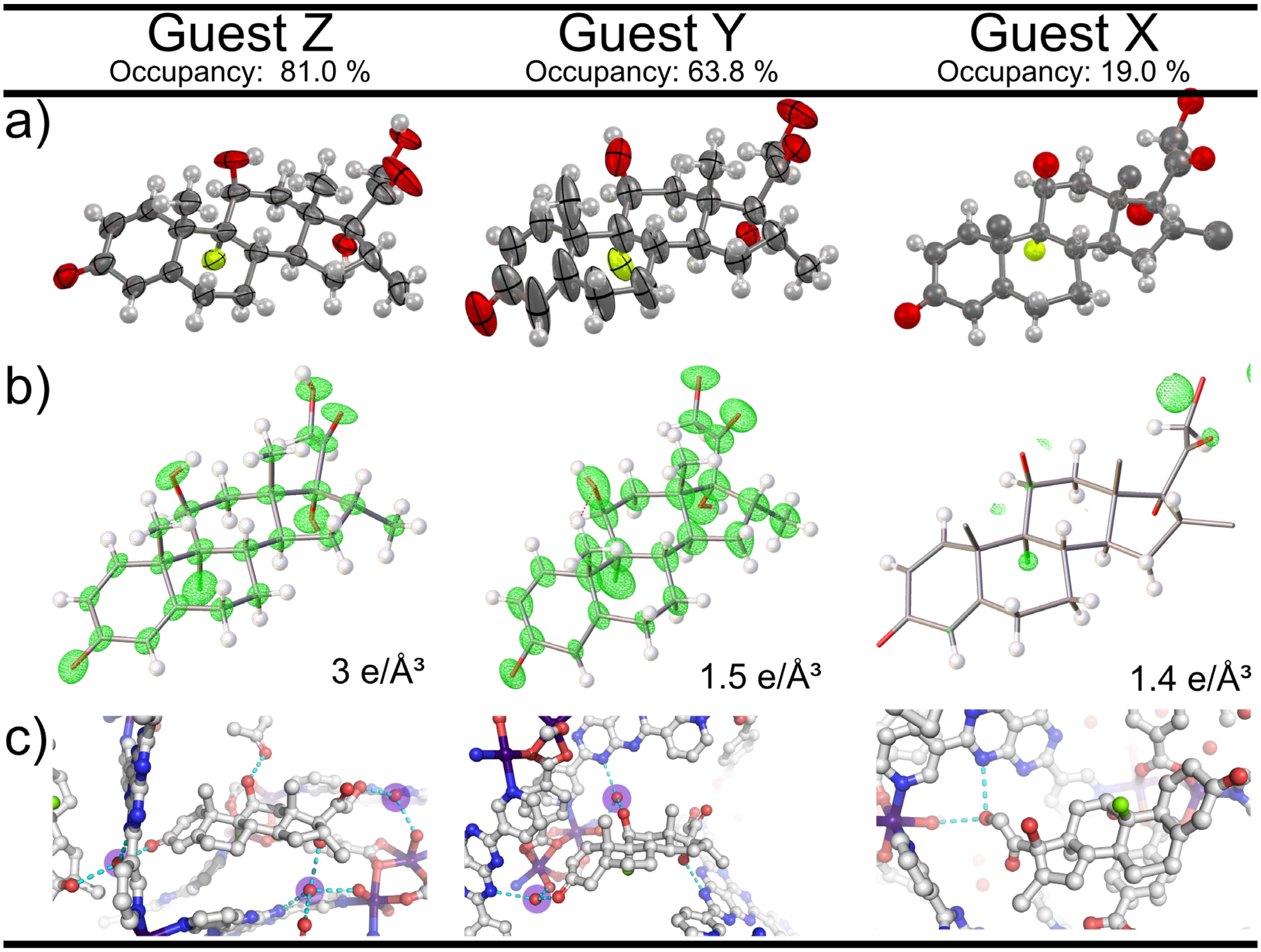

Supplementary Fig 5.
Dexamethasone encapsulation summary. a) isotropic and anisotropic models (ORTEP figure) with 50% probability, b) 2Fo-Fc electron density map with the number showing the map threshold, c) hydrogen bonding interactions between Co-3TPHAP, water and dexamethasone. The water molecules highlighted in purple were freely residing inside the pore, cyan dash lines represent hydrogen bond contacts. Atom coloring scheme: a, b) C: grey, O: red, F: yellow-green, and hydrogen: white; c) C: white, N: blue, O: red, F: yellow-green and Co: purple, hydrogen atoms were omitted for clarity.

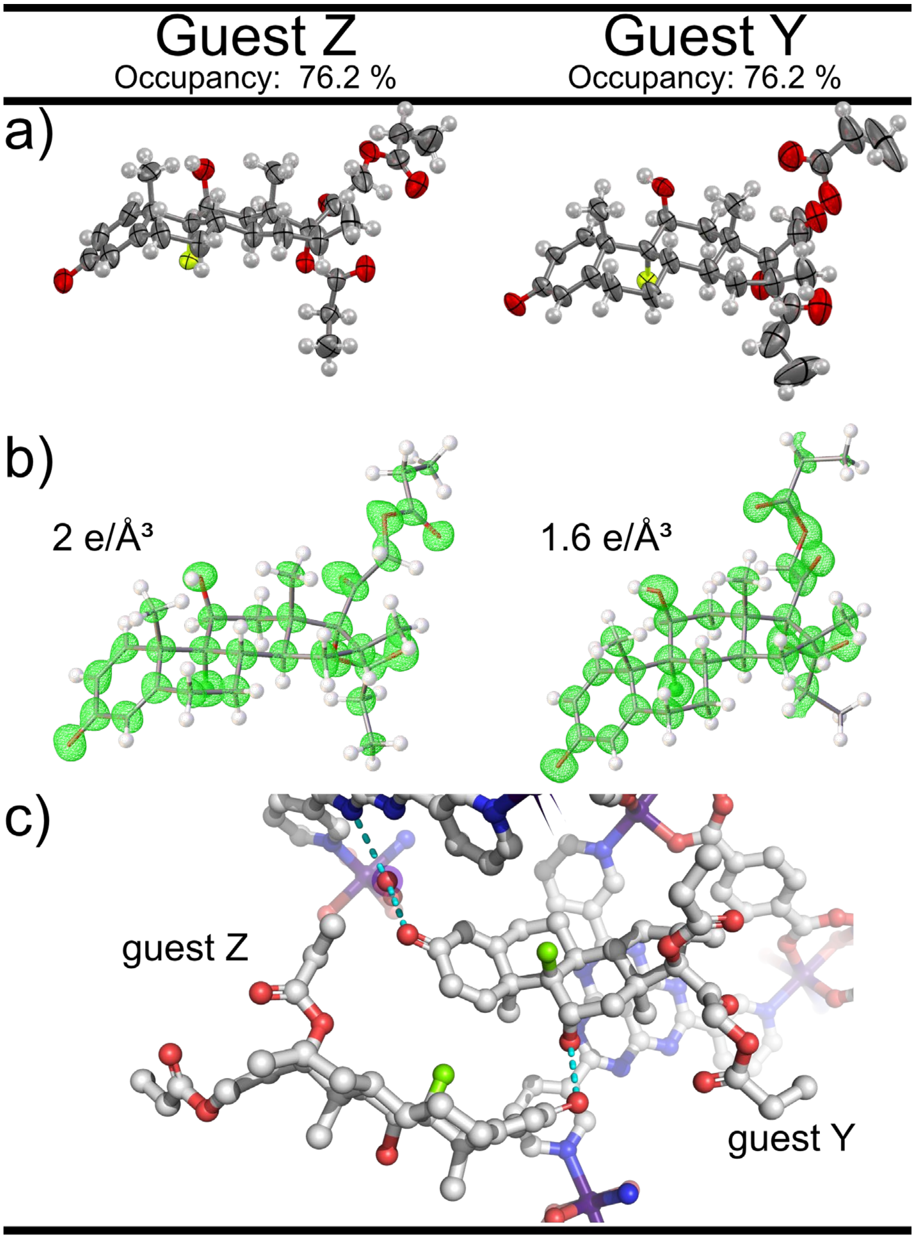

Supplementary Fig 6.
Betamethasone dipropionate encapsulation summary. a) ORTEP figure with 50% probability, b) 2Fo-Fc electron density map with the number showing the map threshold, c) hydrogen bonding interactions between Co-3TPHAP and betamethasone dipropionate. Atom coloring scheme: a, b) C: grey, O: red, F: yellow-green, and hydrogen: white; c) C: white, N: blue, O: red, F: yellow-green and Co: purple, hydrogen atoms were omitted for clarity.

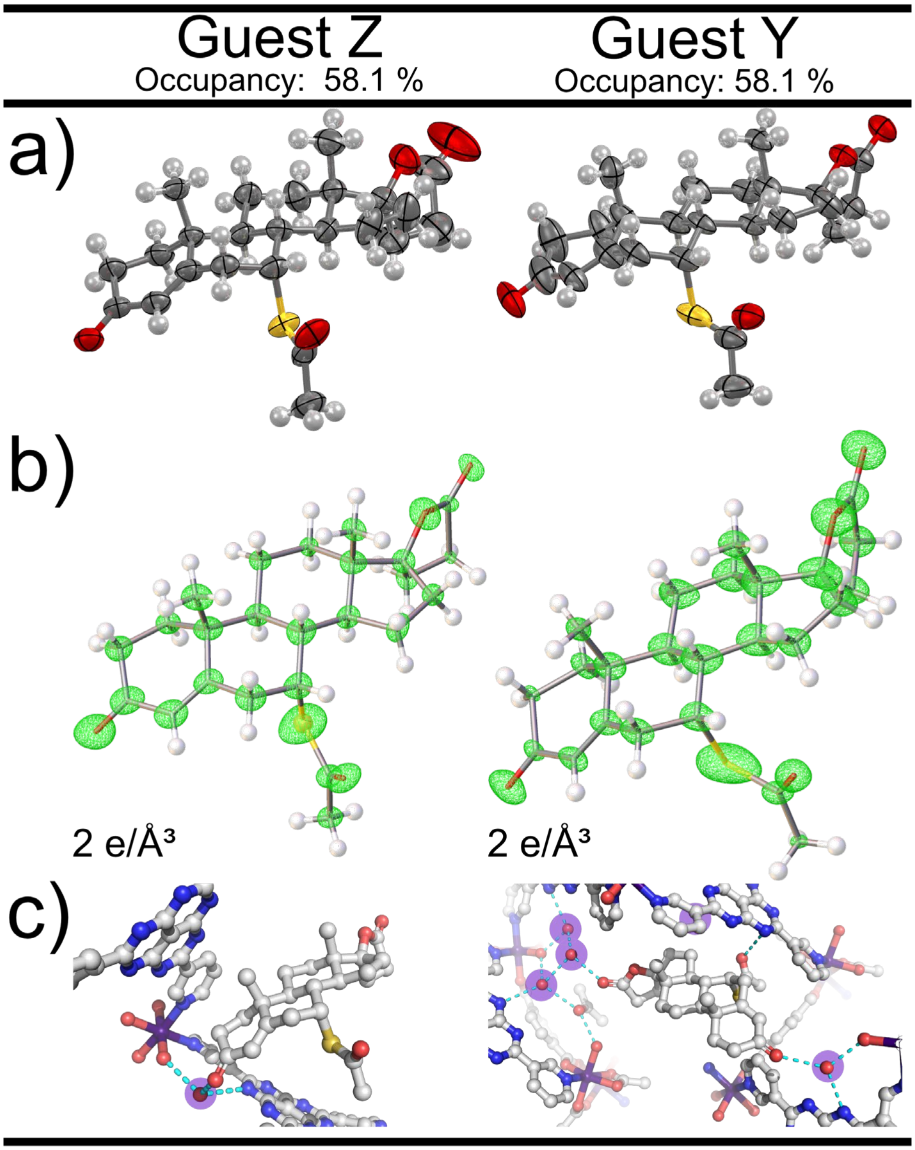

Supplementary Fig 7.
Spironolactone encapsulation summary. a) ORTEP figure with 50% probability, b) 2Fo-Fc electron density map with the number showing the map threshold, c) hydrogen bonding interactions between Co-3TPHAP, water and spironolactone. The water molecules highlighted in purple were freely residing inside the pore, cyan dash lines represent hydrogen bond contacts. Atom coloring scheme: a, b) C: grey, O: red, S: yellow, and hydrogen: white; c) C: white, N: blue, O: red, S: yellow and Co: purple, hydrogen atoms were omitted for clarity.

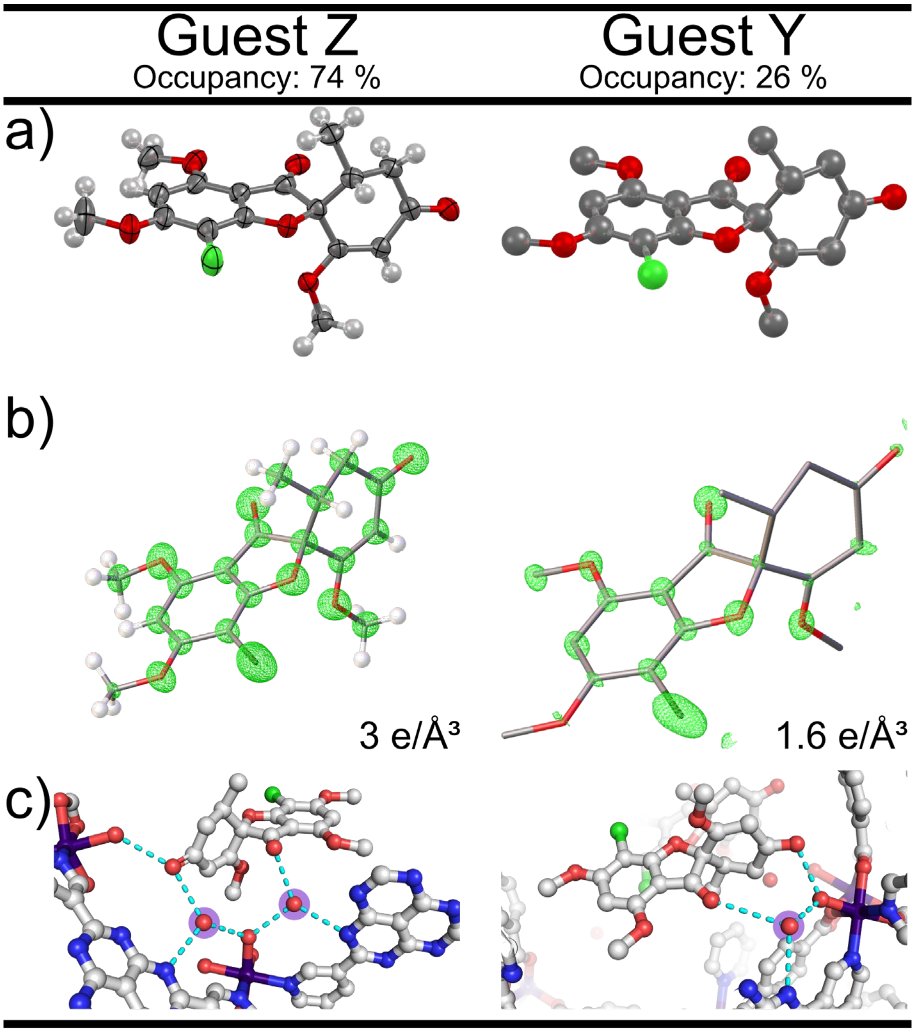

Supplementary Fig 8.
(+)-griseofulvin encapsulation summary. a) isotropic and anisotropic models (ORTEP figure) with 50% probability, b) 2Fo-Fc electron density map with the number showing the map threshold, c) hydrogen bonding interactions between Co-3TPHAP, water and (+)-griseofulvin. The water molecules highlighted in purple were freely residing inside the pore, cyan dash lines represent hydrogen bond contacts. Atom coloring scheme: a, b) C: grey, O: red, Cl: green, and hydrogen: white; c) C: white, N: blue, O: red, Cl: green and Co: purple, hydrogen atoms were omitted for clarity.

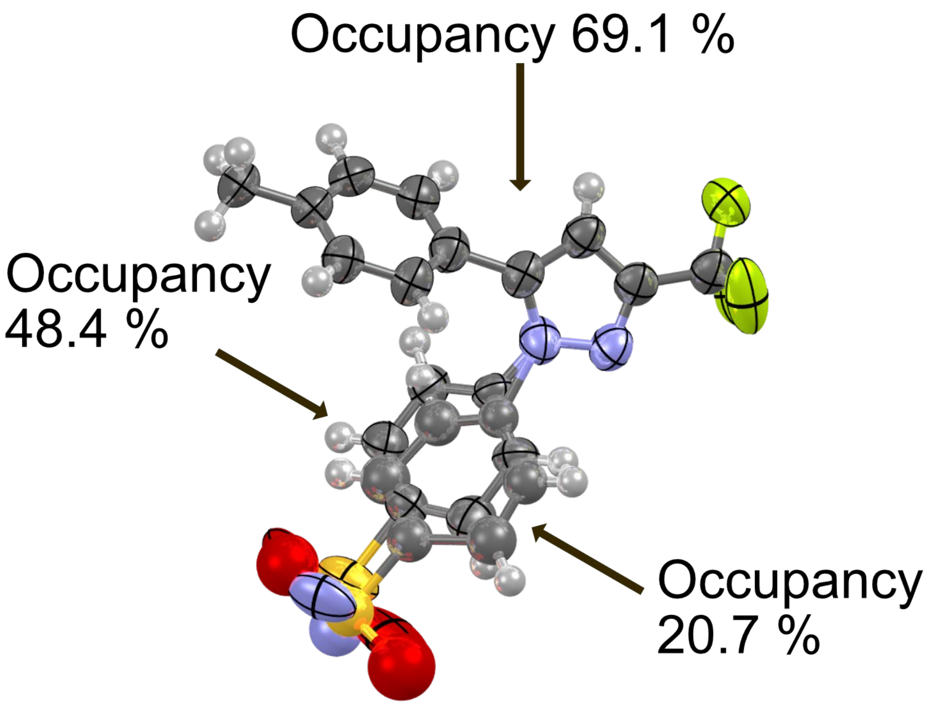

Supplementary Fig 9.
The structure of celecoxib encapsulated inside Co-3TPHAP. Models containing isotropic and anisotropic atoms (ORTEP figure) with 50% probability. Two disordered molecules are shown. Atom coloring scheme: C: grey, N: blue, O: red, S: yellow, F: yellow-green, and hydrogen: white.

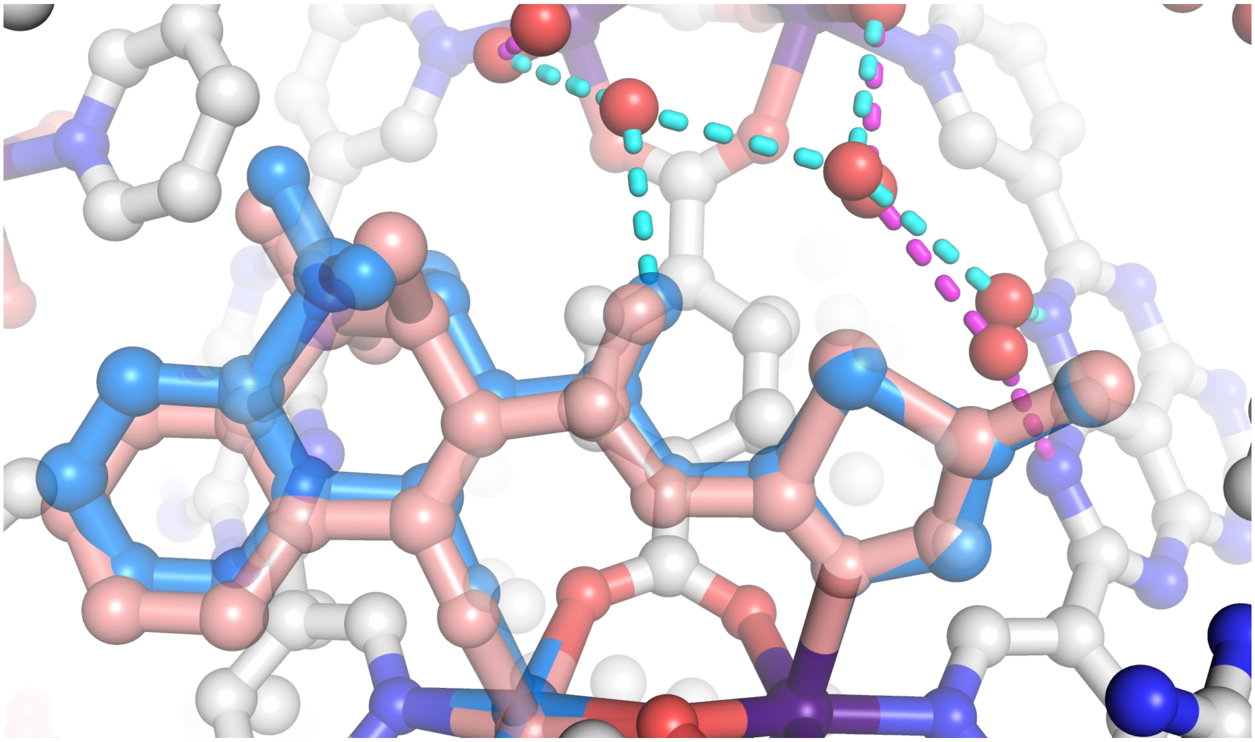

Supplementary Fig 10

The disorder model of meloxicam inside Co-3TPHAP. Blue (part 1) has 64.8% occupancy and pink (part 2) has 35.2% occupancy. The cyan dash lines are showing hydrogen bonding of part 1, and magenta dash lines are hydrogen bonding of part 2. Atom coloring scheme: C: white, N: blue, O: red and Co: purple, hydrogen atoms were omitted for clarity.

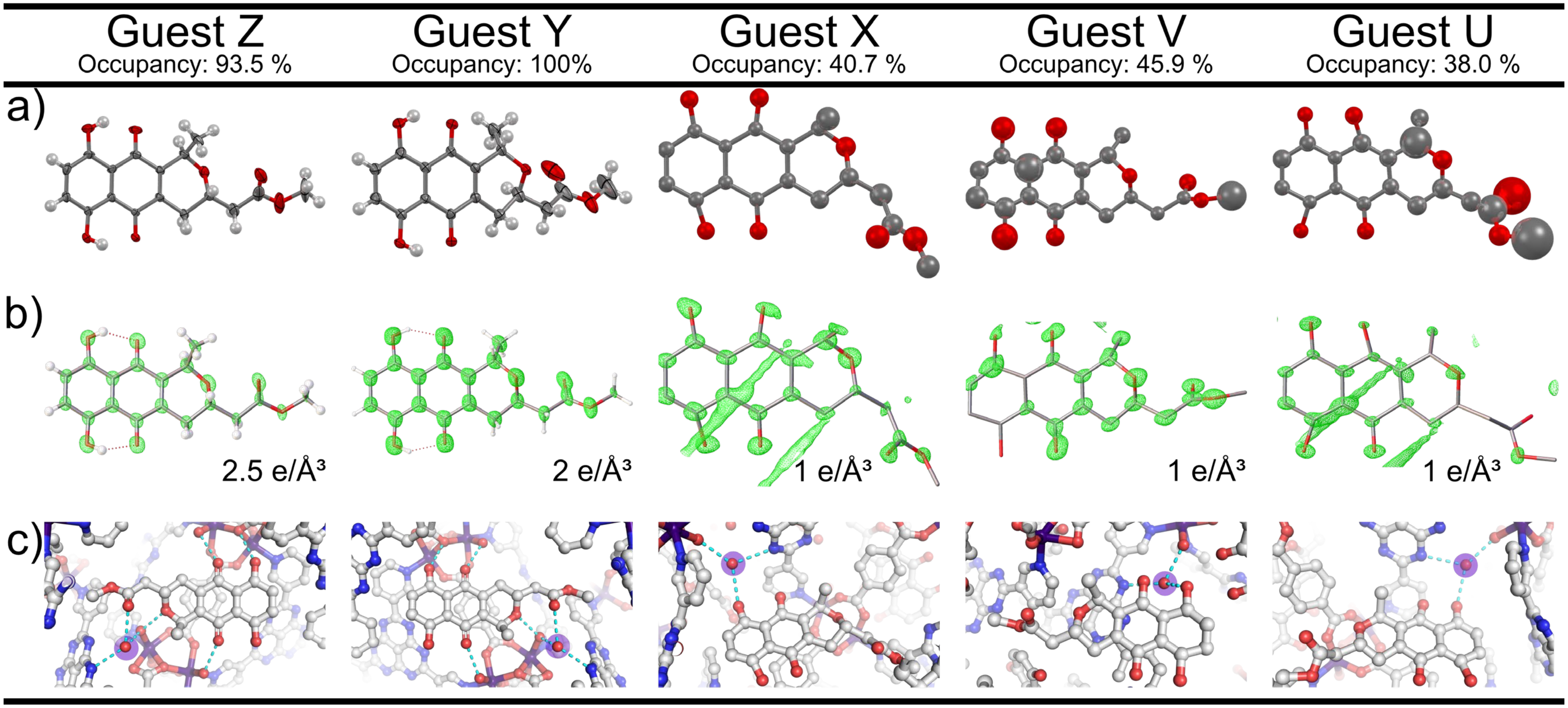

Supplementary Fig 11.
Hemi-actinorhodin methyl ester encapsulation summary. a) Isotropic and anisotropic models (ORTEP figure) with 50% probability, b) 2Fo-Fc electron density map with the number showing the map threshold, c) hydrogen bonding interactions between Co-3TPHAP, water and hemi-actinorhodin methyl ester. The water molecules highlighted in purple were freely residing inside the pore, cyan dash lines represent hydrogen bond contacts. Atom coloring scheme: a, b) C: grey, O: red, and hydrogen: white; c) C: white, N: blue, O: red, and Co: purple, hydrogen atoms were omitted for clarity.

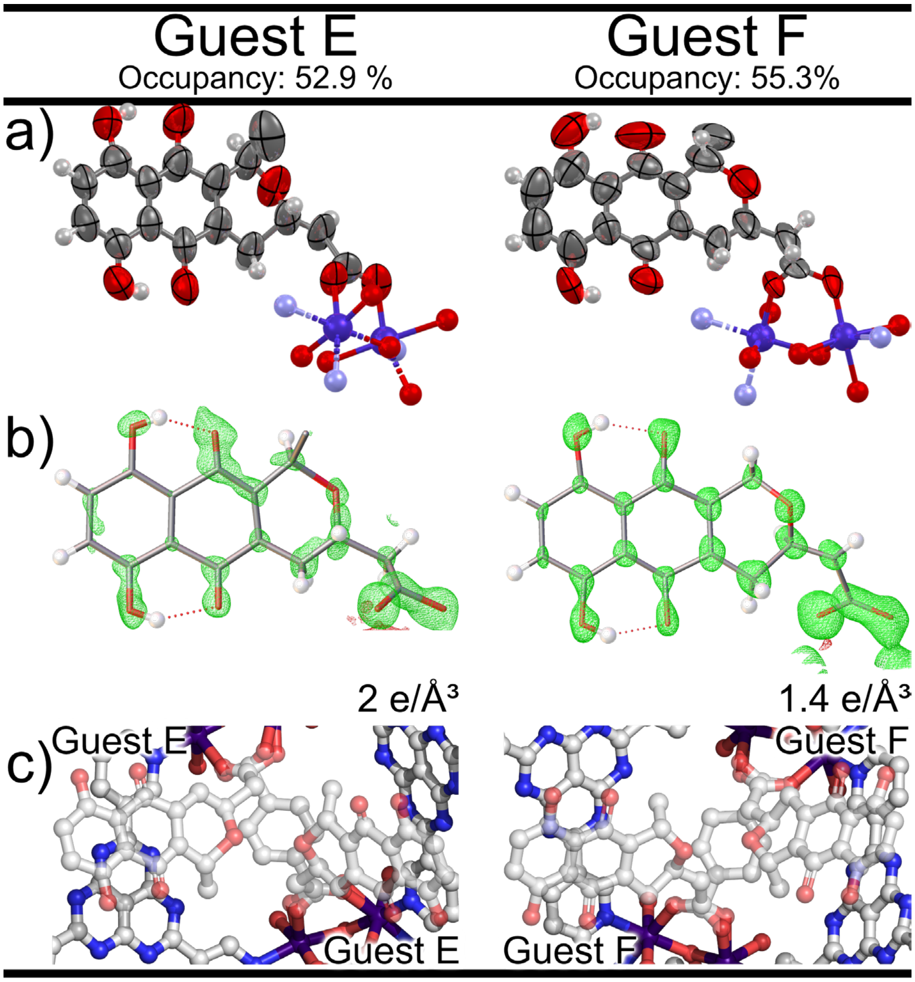

Supplementary Fig 12.
Hemi-actinorhodin encapsulation summary. a) ORTEP figure with 50% probability, b) 2Fo-Fc electron density map with the number showing the map threshold, c) disorder models between hemi-actinorhodin and terephthalate. Atom coloring scheme: a, b) C: grey, N: blue, O: red, Co: purple, and hydrogen: white; c) C: white, N: blue, O: red, and Co: purple, hydrogen atoms were omitted for clarity.

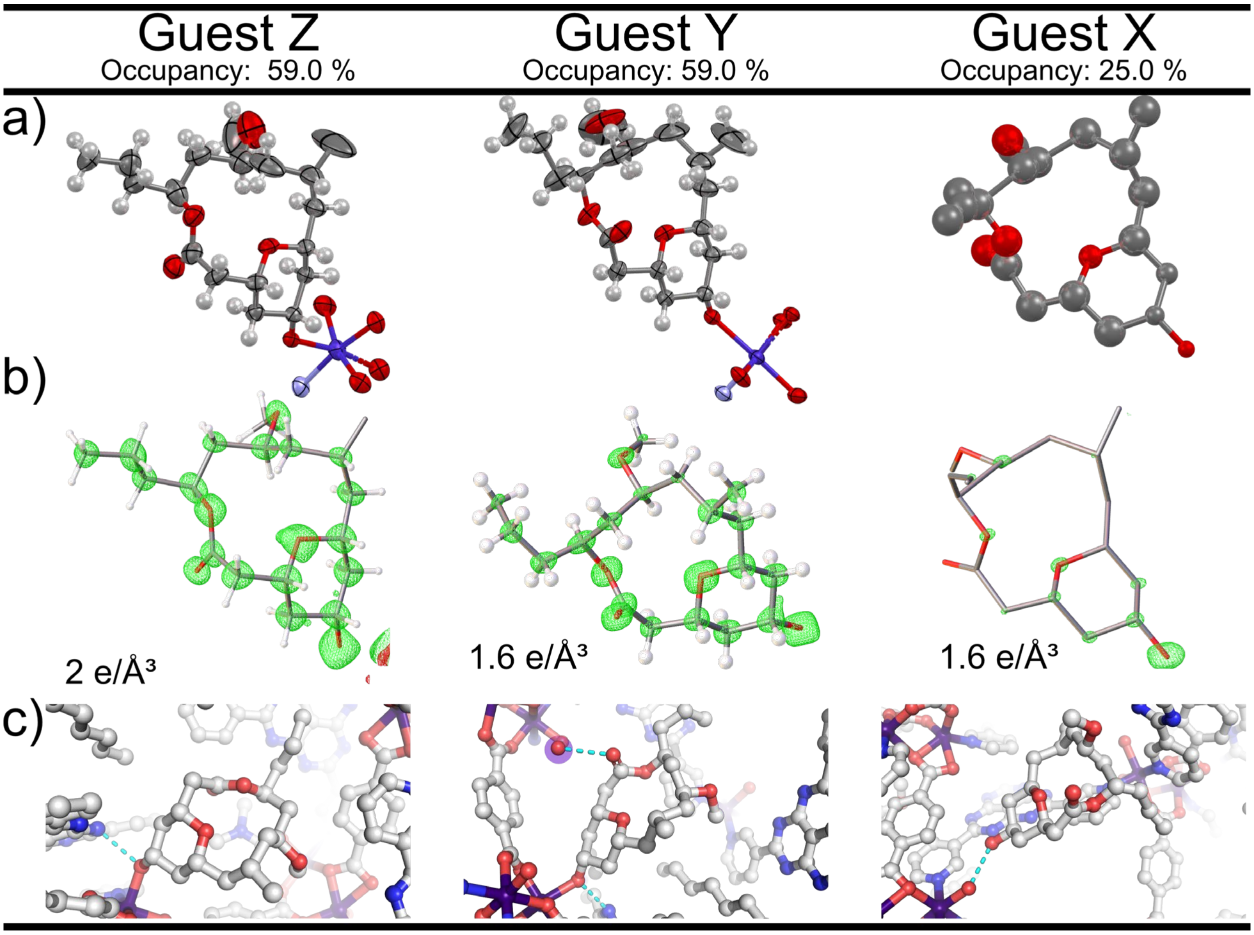

Supplementary Fig 13.
Neopeltolide macrolactone encapsulation summary. a) ORTEP figure with 50% probability, b) 2Fo-Fc electron density map with the number showing the map threshold, c) hydrogen bonding interactions between Co-3TPHAP, water and neopeltolide macrolactone. The water molecules highlighted in purple were freely residing inside the pore, cyan dash lines represent hydrogen bond contacts. Atom coloring scheme: a, b) C: grey, N: blue, O: red, Co: purple, and hydrogen: white; c) C: white, N: blue, O: red, and Co: purple, hydrogen atoms were omitted for clarity.

a)

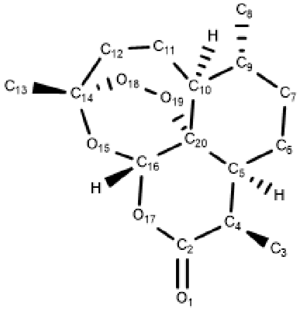

b)

Flack parameter	Artemisinin crystal	Co-3TPHAP
	0.02(6)	0.022(1)

c)

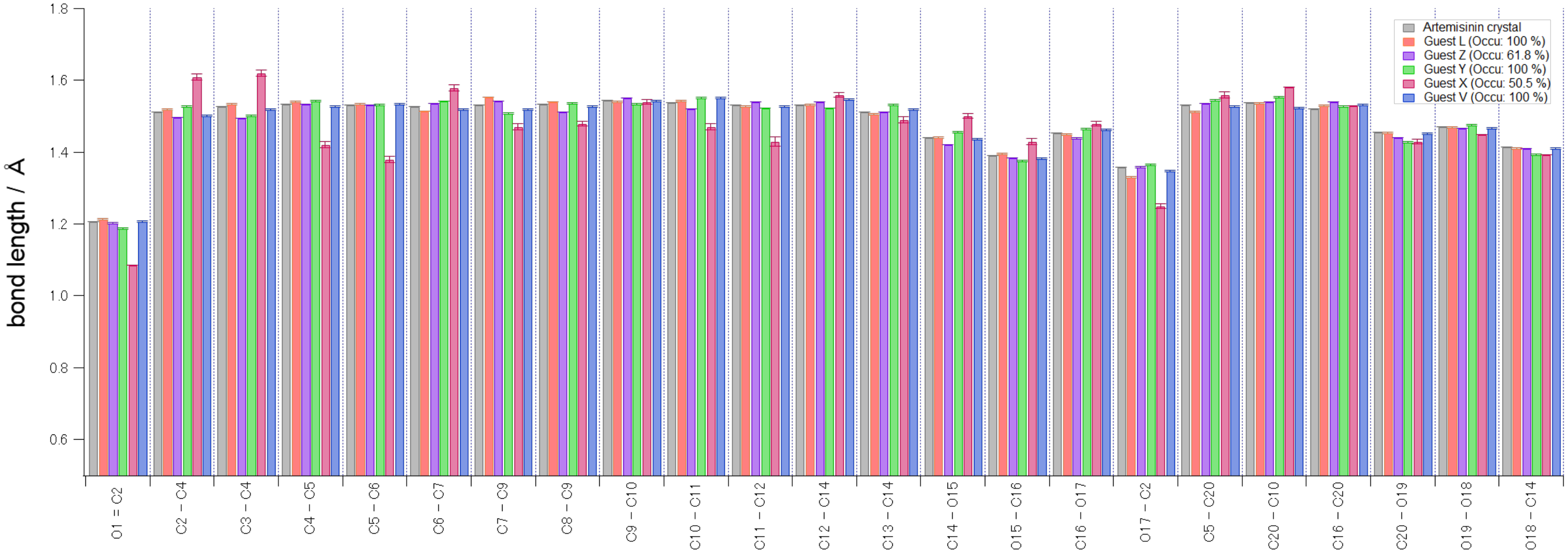

Supplementary Fig 14.
Comparison of artemisinin structural parameters obtained from a pure single crystal and after encapsulation into Co-3TPHAP; a) atom numbering scheme, b) Flack parameters, c) summary of bond lengths with the corresponding error bars. The error bars were obtained from the refinement error by SHELXL-2018.

a)

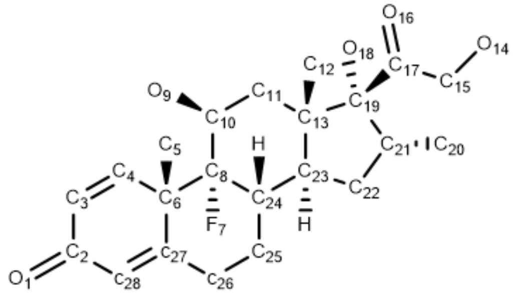

b)

Flack parameter	Dexamethasone crystal	Co-3TPHAP
	-0.02(5)	0.053(2)

c)

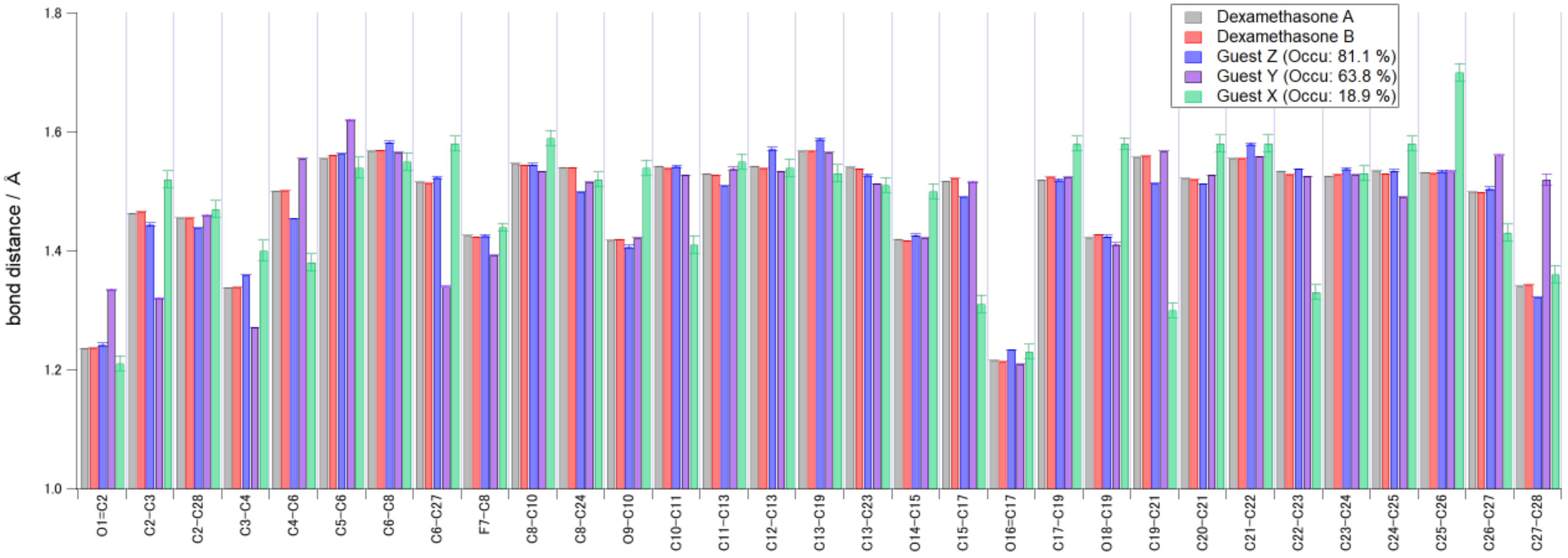

Supplementary Fig 15.
Comparison of dexamethasone structural parameters obtained from a pure single crystal and after encapsulation into Co-3TPHAP; a) atom numbering scheme, b) Flack parameters, c) summary of bond lengths with the corresponding error bars. The error bars were obtained from the refinement error by SHELXL-2018.

a)

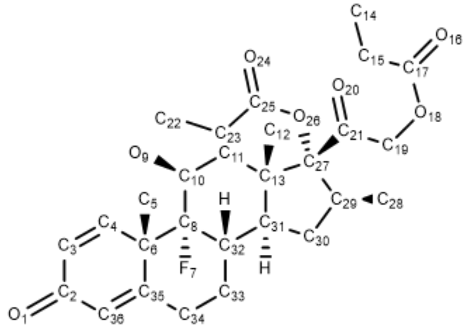

b)

Flack parameter	Betamethasone dipropionate crystal	Co-3TPHAP
	0.06(4)	0.042(2)

c)

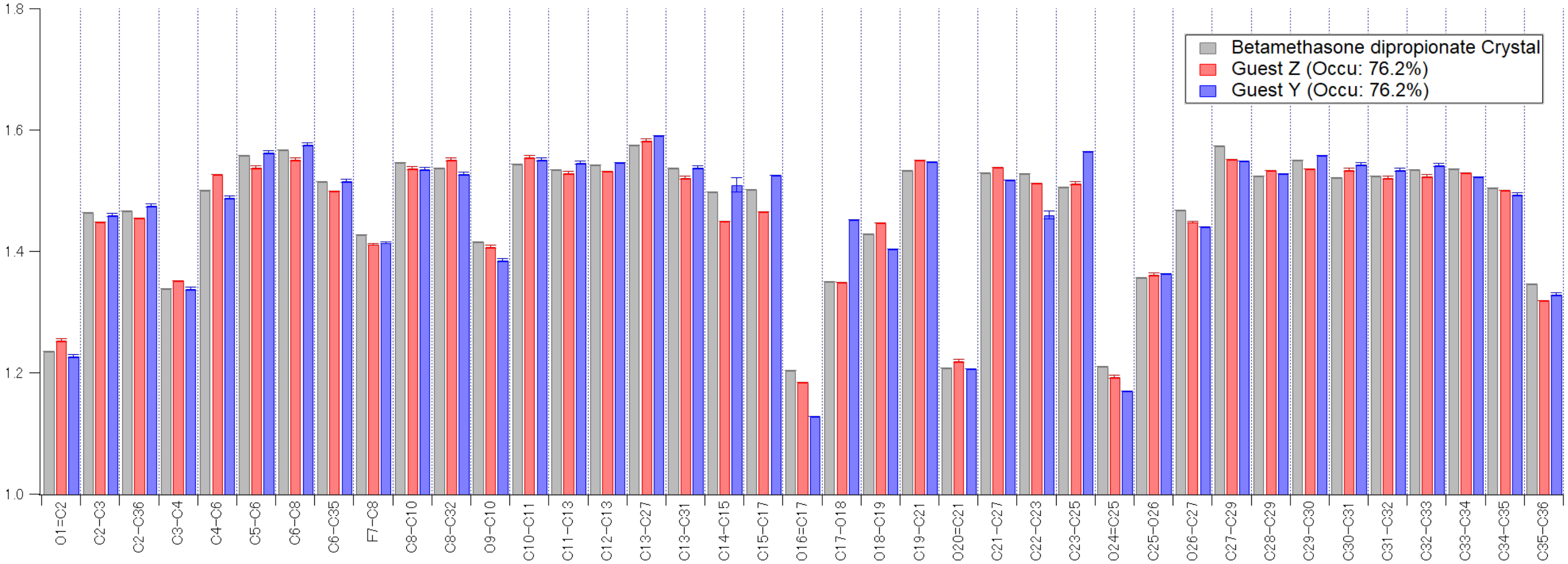

Supplementary Fig 16.
Comparison of betamethasone dipropionate structural parameters obtained from a pure single crystal and after encapsulation into Co-3TPHAP; a) atom numbering scheme, b) Flack parameters, c) summary of bond lengths with the corresponding error bars. The error bars were obtained from the refinement error by SHELXL-2018.

a)

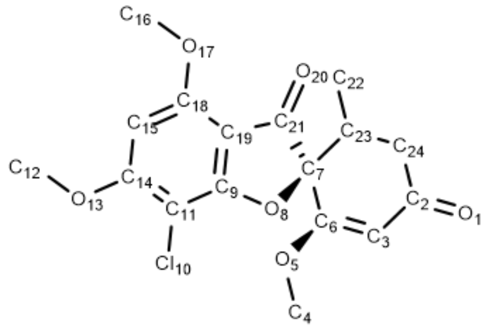

b)

Flack parameter	(+)-Griseofulvin crystal	Co-3TPHAP
	0.021(4)	0.041(2)

c)

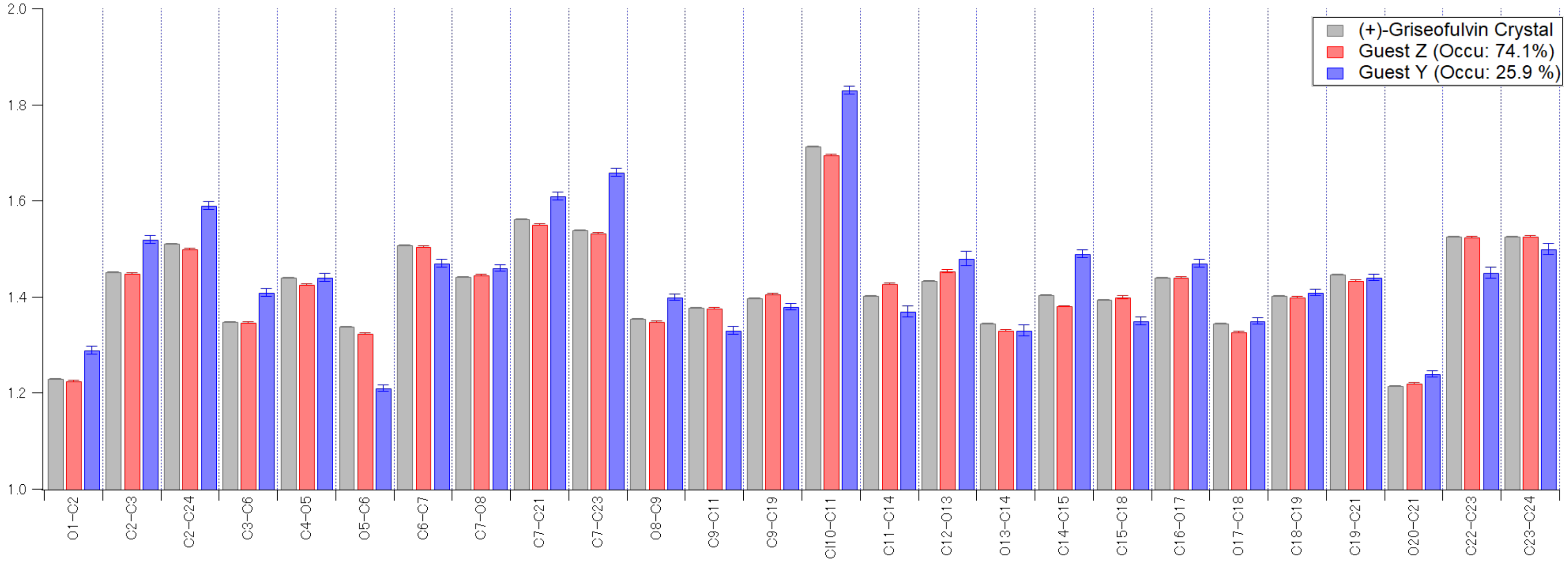

Supplementary Fig 17.
Comparison of (+)-griseofulvin structural parameters obtained from a pure single crystal and after encapsulation into Co-3TPHAP; a) atom numbering scheme, b) Flack parameters, c) summary of bond lengths with the corresponding error bars. The error bars were obtained from the refinement error by SHELXL-2018.

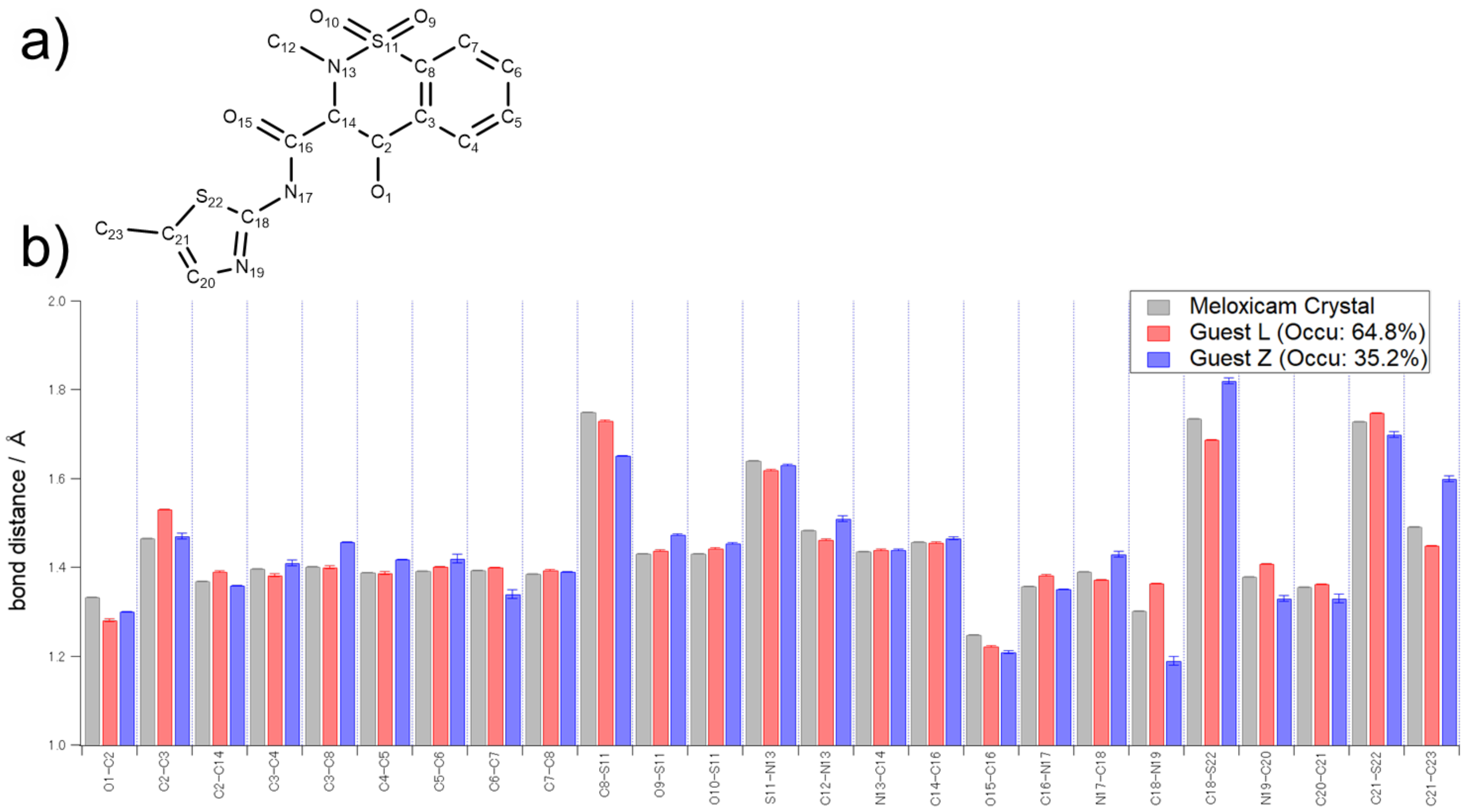

Supplementary Fig 18.
Comparison of meloxicam structural parameters obtained from a pure single crystal and after encapsulation into Co-3TPHAP; a) atom numbering scheme, b) summary of bond lengths with the corresponding error bars. The error bars were obtained from the refinement error by SHELXL-2018.

Supplementary Tab 1.
Guest encapsulation conditions.

Guest (amount)	Encapsulation solvent
Artemisinin (3 mg)	*n*-heptane 1 mL
Artemether (4 mg)	*n*-heptane 1 mL
Dihydroartemisinin (3 mg)	*n*-heptane 1 mL
Artesunate (3 mg)	*n*-heptane 1 mL + acetone 0.1 mL
Dexamethasone (2 mg)	*n*-heptane 1 mL + acetone 0.1 mL
Betamethasone (3 mg)	*n*-heptane 1 mL + acetone 0.1 mL
Betamethasone 17,21-Dipropionate (2 mg)	*n*-heptane 1 mL + acetone 0.1 mL
Spironolactone (2 mg)	*n*-heptane 1 mL + acetone 0.1 mL
(+)-Griseofulvin (2 mg)	*n*-heptane 1 mL + acetone 0.1 mL
Celecoxib (1.6 mg)	*n*-heptane 1 mL + acetone 0.1 mL
Meloxicam (3 mg)	*n*-heptane 1 mL
Hemi-actinorhodin methyl ester (0.5 mg)	*n*-heptane 1 mL
Hemi-actinorhodin (0.5 mg)	*n*-heptane 1 mL + acetone 0.1 mL
Neopeltolide macrolactone (2 mg)	*n*-heptane 0.1 mL

Supplementary Tab 2.
Concentrations of saturated guest solutions (mg mL^{-1}) measured by UPMS. The highlighted cells correspond to the conditions used for encapsulation into Co-3TPHAP.

**Compound name**	***n*-heptane**	**Acetone/*n*-heptane 10 % (v/v)**
Dexamethasone	< 0.00039	0.013
Betamethasone	< 0.00039	0.022
Celecoxib	0.0038	1.621
(+)-Griseofulvin	0.012	0.141
Artesunate	0.013	1.504
Meloxicam	0.016	0.032
Betamethasone dipropionate	0.031	0.98
Spironolactone	0.2	2.32
Dihydroartemisinin	0.266	2.166
Artemisinin	2.795	7.285
Artemether	500 >	-

Supplementary Tab 3.
A list of crystallographic restraints and constraints applied during the structure analysis of encapsulated guest.

**Guest name**		**Restraints and constraints**
Artemisinin	Guest L	**None**
	Guest Z	**None**
	Guest Y	**None**
	Guest X	**None**
	Guest V	**None**
Artemether	Guest Z	**None**
Artesunate	Guest L	**None**
	Guest Z	**None**
Dihydroartemisinin	Guest Z	**None**
	Guest Y	**None**
	Guest X	**None**
	Guest V	SAME O1U > C210U
	Guest U	**None**
Betamethasone	Guest Z	**None**
	Guest Y	**None**
Dexamethasone	Guest Z	**None**
	Guest Y	**None**
	Guest X	**None**
Betamethasone dipropionate	Guest Z	**None**
	Guest Y	**None**
Spironolactone	Guest Z	**None**
	Guest Y	**None**
Griseofulvin	Guest Z	**None**
	Guest Y	**None**
Celecoxib	Guest Z	**None**
	Guest Y	DFIX (S23Y-O26Y)

Guest name		Restraints and constraints
Meloxicam	Guest L	**None**
	Guest Z	**None**
Hemi-actinorhodin methyl ester	Guest Z	**None**
	Guest Y	**None**
	Guest X	SAME O1Z > C24Z
	Guest V	SAME O1Y > C24Y<br>EADP O18V C20V C21V
	Guest U	SAME O1Y > C24Y<br>EADP O18U C20U C21U
Hemi-actinorhodin	Guest E	SIMU C4E > O23E<br>DELU C4E > O23E<br>EADP O1E O2H<br>EADP O2E O1H<br>EADP C3E C3H<br>DFIX 1.23 O1E C3E<br>DFIX 1.23 C3E O1E
	Guest F	SIMU C4F > O23F<br>DELU C4F > O23F<br>EADP O1F O2G<br>EADP O2F O1G<br>EADP C3F C3G<br>DFIX 1.23 C3F O2F<br>DFIX 1.23 C3F O1F<br>DFIX 1.5 C22F C21F
Neopeltolide macrolactone	Guest Z	**None**
	Guest Y	**None**
	Guest X	EADP (C4X > C22X)

Supplementary Tab 4.
Calculations of binding energy for each encapsulated guest.

Guest name	Normalized MOF-Guest binding (A) /kJ mol^{-1}	Normalized Water-Guest binding (B) /kJ mol^{-1}	A + B /kJ mol^{-1}	Normalized MOF-Water-Guest binding /kJ mol^{-1}
Artemisinin	-154.2	-28.9	-183.0	-177.3
Artemether	-124.0	-45.6	-169.7	-161.6
Artesunate	-216.2	-18.4	-234.5	-238.9
Dihydroartemisinin	-122.0	-45.8	-167.8	-168.6
Dexamethasone	-158.4	-57.9	-216.3	-218.1
Betamethasone	-146.3	-35.5	-181.8	-177.4
Betamethasone dipropionate	-214.4	-14.1	-228.4	-228.0
Spironolactone	-176.5	-37.8	-214.3	-210.9
Celecoxib	-137.1	2.9	-134.3	-153.4
Griseofulvin	-175.9	-45.9	-221.8	-216.5
Meloxicam	-421.5	-41.8	-463.3	-479.9

Supplementary Tab 5.
Crystallographic table.

**Identification code**	**artemisinin@Co-3TPHAP**	**artemether@Co-3TPHAP**	**artesunate@Co-3TPHAP**
Empirical formula	$C_{223.68}H_{174.22}Co_{8}N_{36}O_{61.35}$	$C_{86.97}H_{62.52}Co_{4}N_{18}O_{24.71}$	$C_{188.22}H_{139.54}Co_{8}N_{36}O_{59.48}$
Formula weight	4819.42	1990.89	4328.74
Temperature/K	95	95	95
Crystal system	monoclinic	monoclinic	monoclinic
Space group	$P2_1$	$C2$	$P2_1$
a/Å	30.753(4)	31.781(3)	30.012(2)
b/Å	16.862(3)	17.143(1)	17.143(6)
c/Å	31.505(4)	29.748(2)	30.926(3)
α/°	90	90	90
β/°	118.458(2)	118.776(4)	118.849(1)
γ/°	90	90	90
Volume/Å^3	14363(4)	14206(1)	13937(5)
Z	2	4	2
ρ_{calc}g/cm^3	1.114	0.931	1.032
μ/mm^{-1}	0.600	0.591	0.610
F(000)	4950.0	4064.0	4425.0
Crystal size/mm^3	0.1913 × 0.153 × 0.072	0.2 × 0.2 × 0.022	0.08 × 0.055 × 0.04
Radiation	synchrotron (λ = 0.750)	synchrotron (λ = 0.750)	synchrotron (λ = 0.750)
2Θ range for data collection/°	1.552 to 60.458	2.944 to 71.354	1.586 to 71.418
Index ranges	-38 ≤ h ≤ 38, -21 ≤ k ≤ 21, -42 ≤ l ≤ 42	-47 ≤ h ≤ 47, -25 ≤ k ≤ 25, -44 ≤ l ≤ 44	-46 ≤ h ≤ 46, -25 ≤ k ≤ 25, -48 ≤ l ≤ 47
Reflections collected	208817	154533	311064
Independent reflections	67757 [R_{int} = 0.0203, R_{sigma} = 0.0198]	47554 [R_{int} = 0.0311, R_{sigma} = 0.0293]	89914 [R_{int} = 0.0273, R_{sigma} = 0.0245]
Data/restraints/parameters	67757/1/3367	47548/1/1330	89914/86/2956
Goodness-of-fit on F^2	1.053	1.038	1.048
Final R indexes [I>=2σ (I)]	R_1 = 0.0548, wR_2 = 0.1630	R_1 = 0.0688, wR_2 = 0.2007	R_1 = 0.0654, wR_2 = 0.1951
Final R indexes [all data]	R_1 = 0.0552, wR_2 = 0.1637	R_1 = 0.0745, wR_2 = 0.2109	R_1 = 0.0717, wR_2 = 0.2033
Largest diff. peak/hole / e Å^{-3}	1.22/-0.75	0.90/-1.18	0.95/-0.96
Flack parameter	0.021(1)	0.019(2)	0.049(2)
CCDC	2246425	2246426	2246427

Identification code	dihydroartemisinin@Co-3TPHAP	dexamethasone@Co-3TPHAP	betamethasone@Co-3TPHAP
Empirical formula	$C_{99.64}H_{73.28}Co_4N_{18.41}O_{29.5}$	$C_{93.58}H_{64.66}Co_4F_{0.82}N_{18}O_{28.08}$	$C_{91.08}H_{53.3}Co_4F_{0.5}N_{18}O_{25.6}$
Formula weight	2236.25	2141.86	2054.84
Temperature/K	95	95	95
Crystal system	monoclinic	monoclinic	monoclinic
Space group	$P2_1$	$P2_1$	$P2_1$
a/Å	29.9570(15)	26.899(3)	27.657(5)
b/Å	17.233(2)	18.352(3)	18.279(2)
c/Å	30.7940(7)	28.460(2)	28.575(2)
α/°	90	90	90
β/°	118.633(1)	115.192(5)	115.940(8)
γ/°	90	90	90
Volume/Å^3	13953(2)	12713(2)	12990(3)
Z	4	4	4
ρ_{calc}g/cm^3	1.065	1.119	1.051
μ/mm^{-1}	0.612	0.668	0.650
F(000)	4576.0	4369.0	4173.0
Crystal size/mm^3	0.133 × 0.07 × 0.05	0.12 × 0.12 × 0.06	0.2 × 0.2 × 0.03
Radiation	synchrotron (λ = 0.750)	synchrotron (λ = 0.750)	synchrotron (λ = 0.750)
2Θ range for data collection/°	1.634 to 71.404	1.668 to 71.388	1.672 to 71.35
Index ranges	-46 ≤ h ≤ 46, -25 ≤ k ≤ 25, -37 ≤ l ≤ 37	-31 ≤ h ≤ 31, -27 ≤ k ≤ 27, -44 ≤ l ≤ 44	-38 ≤ h ≤ 38, -27 ≤ k ≤ 27, -43 ≤ l ≤ 44
Reflections collected	311289	282265	282853
Independent reflections	89538 [R_{int} = 0.0234, R_{sigma} = 0.0211]	83438 [R_{int} = 0.0203, R_{sigma} = 0.0188]	85131 [R_{int} = 0.0212, R_{sigma} = 0.0206]
Data/restraints/parameters	89534/115/3173	83438/1/3018	85131/1/2775
Goodness-of-fit on F^2	1.041	1.038	1.084
Final R indexes [I>=2σ (I)]	R_1 = 0.0531, wR_2 = 0.1599	R_1 = 0.0707, wR_2 = 0.2135	R_1 = 0.0788, wR_2 = 0.2461
Final R indexes [all data]	R_1 = 0.0548, wR_2 = 0.1627	R_1 = 0.0839, wR_2 = 0.2320	R_1 = 0.0921, wR_2 = 0.2655
Largest diff. peak/hole / e Å^{-3}	1.01/-0.71	0.91/-0.89	0.84/-1.06
Flack parameter	0.019(1)	0.053(2)	0.111(3)
CCDC	2246428	2246429	2246430

Identification code	betamethasone dipropionate @Co-3TPHAP	spironolactone@Co-3TPHAP	(+)-griseofulvin@Co-3TPHAP
Empirical formula	$C_{194.68}H_{139.17}Co_8F_{1.53}N_{36}O_{59.07}$	$C_{183.81}H_{120.99}Co_{7.94}N_{36}O_{53.48}S_{1.18}$	$C_{91.15}H_{56.09}ClCo_4N_{18}O_{28.41}$
Formula weight	4427.64	4195.06	2129.22
Temperature/K	95	95	95
Crystal system	monoclinic	monoclinic	monoclinic
Space group	$P2_1$	$P2_1$	$C2$
a/Å	27.834(3)	28.604(3)	30.865(8)
b/Å	18.284(7)	17.728(1)	18.036(1)
c/Å	28.753(3)	30.022(3)	28.077(1)
α/°	90	90	90
β/°	116.902(3)	116.342(4)	120.122(4)
γ/°	90	90	90
Volume/$Å^3$	13049(5)	13643(8)	13519(4)
Z	2	2	4
ρ_{calc}g/cm^3	1.127	1.021	1.046
μ/mm^{-1}	0.654	0.626	0.650
F(000)	4522.0	4274.0	4325.0
Crystal size/mm^3	0.2 × 0.18 × 0.12	0.2 × 0.14 × 0.11	0.21 × 0.12 × 0.06
Radiation	synchrotron (λ = 0.750)	synchrotron (λ = 0.750)	synchrotron (λ = 0.750)
2Θ range for data collection/°	1.676 to 71.404	1.598 to 71.43	1.77 to 71.314
Index ranges	$-42 \le h \le 43$, $-27 \le k \le 27$, $-39 \le l \le 38$	$-44 \le h \le 44$, $-26 \le k \le 27$, $-40 \le l \le 39$	$-46 \le h \le 46$, $-27 \le k \le 27$, $-41 \le l \le 41$
Reflections collected	288520	294654	148224
Independent reflections	85979 [R_{int} = 0.0172, R_{sigma} = 0.0162]	94793 [R_{int} = 0.0289, R_{sigma} = 0.0301]	47982 [R_{int} = 0.0210, R_{sigma} = 0.0214]
Data/restraints/parameters	85979/1/3192	94788/1/3024	47982/1/1485
Goodness-of-fit on F^2	1.041	0.989	1.037
Final R indexes [I>=2σ (I)]	R_1 = 0.0674, wR_2 = 0.1998	R_1 = 0.0764, wR_2 = 0.2278	R_1 = 0.0702, wR_2 = 0.1988
Final R indexes [all data]	R_1 = 0.0748, wR_2 = 0.2114	R_1 = 0.0935, wR_2 = 0.2471	R_1 = 0.0819, wR_2 = 0.2151
Largest diff. peak/hole / e $Å^{-3}$	0.93/-1.05	0.77/-1.19	1.00/-1.68
Flack parameter	0.042(2)	0.057(3)	0.041(2)
CCDC	2246431	2246432	2246433

Identification code	meloxicam@Co-3TPHAP	celecoxib@Co-3TPHAP	hemiactinorhodin methyl ester @Co-3TPHAP
Empirical formula	$C_{105.01}H_{53.41}Co_4N_{21.35}O_{23.84}S_2$	$C_{101.11}H_{53.82}Co_4F_{2.07}N_{20.38}O_{27.09}S_{0.69}$	$C_{103.25}H_{56.98}Co_4N_{18}O_{31.73}$
Formula weight	2295.43	2293.82	2404.97
Temperature/K	95	95	95
Crystal system	monoclinic	monoclinic	monoclinic
Space group	$P2_1/c$	$P2_1/c$	$P2_1$
a/Å	29.663(1)	28.064(3)	30.4900(7)
b/Å	17.326(4)	18.267(5)	16.7370(1)
c/Å	30.7260(7)	28.105(4)	30.8170(1)
α/°	90	90	90
β/°	118.518(3)	116.477(2)	117.198(2)
γ/°	90	90	90
Volume/Å^3	13875(4)	12897(4)	13987.4(1)
Z	4	4	4
ρ_{calc}g/cm^3	1.099	1.180	1.127
μ/mm^{-1}	0.648	0.678	0.615
F(000)	4655.0	4642.0	4815.0
Crystal size/mm^3	0.2 × 0.1 × 0.05	0.08 × 0.06 × 0.02	0.2 × 0.2 × 0.08
Radiation	synchrotron (λ = 0.750)	synchrotron (λ = 0.750)	synchrotron (λ = 0.750)
2Θ range for data collection/°	1.648 to 71.39	1.71 to 71.424	1.568 to 71.426
Index ranges	-44 ≤ h ≤ 44, -24 ≤ k ≤ 24, -47 ≤ l ≤ 47	-42 ≤ h ≤ 43, -28 ≤ k ≤ 28, -39 ≤ l ≤ 39	-45 ≤ h ≤ 45, -25 ≤ k ≤ 25, -45 ≤ l ≤ 45
Reflections collected	300600	283180	311690
Independent reflections	51240 [R_{int} = 0.0232, R_{sigma} = 0.0156]	47129 [R_{int} = 0.0330, R_{sigma} = 0.0210]	90667 [R_{int} = 0.0142, R_{sigma} = 0.0128]
Data/restraints/parameters	51240/0/1819	47129/1/1705	90667/339/3134
Goodness-of-fit on F^2	1.035	1.036	1.072
Final R indexes [I>=2σ (I)]	R_1 = 0.0626, wR_2 = 0.1918	R_1 = 0.0815, wR_2 = 0.2522	R_1 = 0.0531, wR_2 = 0.1671
Final R indexes [all data]	R_1 = 0.0738, wR_2 = 0.2031	R_1 = 0.1067, wR_2 = 0.2808	R_1 = 0.0556, wR_2 = 0.1725
Largest diff. peak/hole / e Å^{-3}	0.85/-0.87	0.89/-1.07	0.94/-0.73
Flack parameter	-	-	0.4957(1)
CCDC	2246435	2246434	2246417

Identification code	hemi-actinorhodin@Co-3TPHAP	neopeltolide macrolactone @Co-3TPHAP	artemisinin
Empirical formula	$C_{92.81}H_{46.76}Co_4N_{18}O_{39.27}$	$C_{87.39}H_{53.11}Co_4N_{18.31}O_{21.57}$	$C_{15}H_{22}O_5$
Formula weight	2278.11	1940.30	282.32
Temperature/K	95	95	95
Crystal system	monoclinic	monoclinic	orthorhombic
Space group	$C2$	$P2_1$	$P2_12_12_1$
a/Å	31.366(3)	28.5230(12)	6.304(3)
b/Å	17.760(3)	17.8490(15)	9.2850(16)
c/Å	28.838(3)	29.8470(7)	23.9410(19)
α/°	90	90	90
β/°	122.814(2)	117.6350(10)	90
γ/°	90	90	90
Volume/Å^3	13501(3)	13461.8(13)	1401.3(8)
Z	4	4	4
ρ_{calc}g/cm^3	1.121	0.957	1.338
μ/mm^{-1}	0.640	0.620	0.112
F(000)	4607.0	3944.0	608.0
Crystal size/mm^3	0.2 × 0.2 × 0.05	0.182 × 0.164 × 0.08	0.25 × 0.25 × 0.1
Radiation	synchrotron (λ = 0.750)	synchrotron (λ = 0.750)	synchrotron (λ = 0.750)
2Θ range for data collection/°	1.774 to 71.366	1.7 to 71.416	4.966 to 71.334
Index ranges	-48 ≤ h ≤ 48, -27 ≤ k ≤ 27, -40 ≤ l ≤ 40	-44 ≤ h ≤ 44, -26 ≤ k ≤ 26, -34 ≤ l ≤ 34	-7 ≤ h ≤ 7, -13 ≤ k ≤ 13, -35 ≤ l ≤ 35
Reflections collected	149264	297395	30306
Independent reflections	48668 [R_{int} = 0.0257, R_{sigma} = 0.0277]	86589 [R_{int} = 0.0156, R_{sigma} = 0.0145]	4874 [R_{int} = 0.0250, R_{sigma} = 0.0153]
Data/restraints/parameters	48668/443/1701	86589/12/2934	4874/0/184
Goodness-of-fit on F^2	1.024	1.032	1.094
Final R indexes [I>=2σ (I)]	R_1 = 0.0960, wR_2 = 0.2680	R_1 = 0.0642, wR_2 = 0.1898	R_1 = 0.0270, wR_2 = 0.0758
Final R indexes [all data]	R_1 = 0.1638, wR_2 = 0.3432	R_1 = 0.0708, wR_2 = 0.1992	R_1 = 0.0272, wR_2 = 0.0760
Largest diff. peak/hole / e Å^{-3}	0.76/-1.00	0.79/-1.02	0.26/-0.21
Flack parameter	0.497(5)	0.059(2)	0.02(6)
CCDC	2246418	2246419	2246420

Identification code	dexamethasone	betamethasone dipropionate
Empirical formula	$C_{22}H_{29}FO_5$	$C_{28}H_{37}FO_7$
Formula weight	392.45	504.57
Temperature/K	95	95
Crystal system	orthorhombic	orthorhombic
Space group	$P2_12_12_1$	$P2_12_12_1$
a/Å	10.328(2)	11.641(1)
b/Å	16.1030(5)	13.7680(5)
c/Å	23.2090(6)	15.5970(8)
α/°	90	90
β/°	90	90
γ/°	90	90
Volume/Å^3	3859.9(7)	2499.8(3)
Z	8	4
ρ_{calc}g/cm^3	1.351	1.341
μ/mm^{-1}	0.113	0.112
F(000)	1680.0	1080.0
Crystal size/mm^3	0.2 × 0.1 × 0.05	0.2 × 0.2 × 0.08
Radiation	synchrotron (λ = 0.750)	synchrotron (λ = 0.750)
2Θ range for data collection/°	3.248 to 71.39	4.164 to 71.406
Index ranges	$-15 \le h \le 15$, $-25 \le k \le 24$, $-34 \le l \le 34$	$-18 \le h \le 18$, $-21 \le k \le 21$, $-23 \le l \le 23$
Reflections collected	85123	54552
Independent reflections	14591 [R_{int} = 0.0184, R_{sigma} = 0.0123]	9564 [R_{int} = 0.0174, R_{sigma} = 0.0110]
Data/restraints/parameters	14591/0/517	9564/0/331
Goodness-of-fit on F^2	1.045	1.060
Final R indexes [I>=2σ (I)]	R_1 = 0.0314, wR_2 = 0.0859	R_1 = 0.0270, wR_2 = 0.0762
Final R indexes [all data]	R_1 = 0.0327, wR_2 = 0.0870	R_1 = 0.0274, wR_2 = 0.0766
Largest diff. peak/hole / e Å^{-3}	0.30/-0.28	0.80/-0.26
Flack parameter	-0.02(5)	0.06(4)
CCDC	2246421	2246422

**Identification code**	**meloxicam**	**(+)-griseofulvin**
Empirical formula	$C_7H_{6.5}N_{1.5}O_2S$	$C_{17}H_{17}ClO_6$
Formula weight	175.70	352.75
Temperature/K	95	95
Crystal system	triclinic	tetragonal
Space group	*P*-1	$P4_1$
a/Å	6.8740(5)	8.8820(3)
b/Å	8.0570(3)	8.8820(3)
c/Å	13.4930(4)	19.5980(4)
α/°	86.193(2)	90
β/°	88.544(2)	90
γ/°	75.467(4)	90
Volume/Å^3	721.76(6)	1546.1(1)
Z	4	4
ρ_{calc}g/cm^3	1.617	1.515
μ/mm^{-1}	0.454	0.321
F(000)	364.0	736.0
Crystal size/mm^3	0.075 × 0.05 × 0.04	0.15 × 0.1 × 0.06
Radiation	synchrotron (λ = 0.750)	synchrotron (λ = 0.750)
2Θ range for data collection/°	3.192 to 71.348	4.84 to 71.224
Index ranges	$-10 \leq h \leq 9$, $-11 \leq k \leq 11$, $-20 \leq l \leq 20$	$-13 \leq h \leq 13$, $-13 \leq k \leq 13$, $-22 \leq l \leq 21$
Reflections collected	7993	34081
Independent reflections	4523 [R_{int} = 0.0148, R_{sigma} = 0.0208]	5014 [R_{int} = 0.0177, R_{sigma} = 0.0103]
Data/restraints/parameters	4523/0/215	5012/1/221
Goodness-of-fit on F^2	1.077	1.076
Final R indexes [I>=2σ (I)]	R_1 = 0.0251, wR_2 = 0.0740	R_1 = 0.0219, wR_2 = 0.0634
Final R indexes [all data]	R_1 = 0.0255, wR_2 = 0.0745	R_1 = 0.0219, wR_2 = 0.0634
Largest diff. peak/hole / e Å^{-3}	0.49/-0.47	0.24/-0.47
Flack parameter	-	0.021(4)
CCDC	2246423	2246424

A- or B-level CheckCif alerts and their responses.

<u>CCDC 2246425 (artemisinin@Co-3TPHAP)</u>

_vrf_PLAT601_Artemisinin@Co-3TPHAP
;
PROBLEM:
PLAT601_ALERT_2_A Unit Cell Contains Solvent Accessible VOIDS of . 294 Ang**3

RESPONSE:
We assigned solvents inside the pore as much as possible.
however, several solvents could not be modeled due to severe disorder.
;

_vrf_PLAT306_Artemisinin@Co-3TPHAP
;
PROBLEM:
PLAT306_ALERT_2_B Isolated Oxygen Atom (H-atoms Missing ?) .... O2W Check
PLAT306_ALERT_2_B Isolated Oxygen Atom (H-atoms Missing ?) .... O5W Check
PLAT306_ALERT_2_B Isolated Oxygen Atom (H-atoms Missing ?) .... O6W Check
PLAT306_ALERT_2_B Isolated Oxygen Atom (H-atoms Missing ?) .... O8W Check
PLAT306_ALERT_2_B Isolated Oxygen Atom (H-atoms Missing ?) .... O9W Check
PLAT306_ALERT_2_B Isolated Oxygen Atom (H-atoms Missing ?) .... O10W Check
PLAT306_ALERT_2_B Isolated Oxygen Atom (H-atoms Missing ?) .... O11W Check
PLAT306_ALERT_2_B Isolated Oxygen Atom (H-atoms Missing ?) .... O13W Check

RESPONSE:
These oxygen atoms are components of the water in the pore of the framework.
Since it was difficult to determine static position of the hydrogen atoms of
the water without clear interactions, the model have no hydrogens.
;

_vrf_PLAT420_Artemisinin@Co-3TPHAP

;
PROBLEM:
PLAT420_ALERT_2_B D-H Bond Without Acceptor O1LD --H1LH . Please Check

RESPONSE:
The hydrogen acceptors were not observed clearly due to severely disorder.
;

_vrf_PLAT430_Artemisinin@Co-3TPHAP
;
PROBLEM:
PLAT430_ALERT_2_B Short Inter D...A Contact O2W ..N8B . 2.84 Ang.
1-x,1/2+y,1-z = 2_656 Check
PLAT430_ALERT_2_B Short Inter D...A Contact O5W ..O10W . 2.82 Ang.
2-x,1/2+y,1-z = 2_756 Check
PLAT430_ALERT_2_B Short Inter D...A Contact O8W ..N8D . 2.84 Ang.
2-x,1/2+y,2-z = 2_757 Check
PLAT430_ALERT_2_B Short Inter D...A Contact O9W ..O10W . 2.71 Ang.
2-x,1/2+y,1-z = 2_756 Check
PLAT430_ALERT_2_B Short Inter D...A Contact O13W ..N20A . 2.87 Ang.
x,y,z = 1_555 Check
RESPONSE:
These interactions should be hydrogen bonds;
however, the hydrogen of waters could not be molded due to disorder, these alerts are inevitable.
;

CCDC 2246426 (artemether@Co-3TPHAP)

_vrf_PLAT602_Artemether@Co-3TPHAP
;
PROBLEM:
PLAT602_ALERT_2_A Solvent Accessible VOID(S) in Structure .... ! Check

RESPONSE:

We assigned solvents inside the pore as much as possible.
however, several solvents could not be modeled due to severe disorder.
;

_vrf_PLAT029_Artemether@Co-3TPHAP
;
PROBLEM:
PLAT029_ALERT_3_B _diffrn_measured_fraction_theta_full value Low . 0.952 Why?

RESPONSE:
In this measurement, the crystal was measured by omega scan.
Omega scan measurement provided the best data quality and it is suitable for this research.
However, diffractiond data set of this crystal was slightly not enough to cover full sphere.
;

_vrf_PLAT049_Artemether@Co-3TPHAP
;
PROBLEM:
PLAT049_ALERT_1_B Calculated Density Less Than 1.0 gcm-3 .... 0.9266 Check

RESPONSE:
This alert is related to PLAT602_ALERT_2_A.
In this analysis, the crystal density appears small due to the presence of voids
that should be occupied by solvent molecules.
;

_vrf_PLAT306_Artemether@Co-3TPHAP
;
PROBLEM:
PLAT306_ALERT_2_B Isolated Oxygen Atom (H-atoms Missing ?) .... O3W Check

PLAT306_ALERT_2_B Isolated Oxygen Atom (H-atoms Missing ?) .... O13W Check

RESPONSE:
These oxygen atoms are components of the water in the pore of the framework.
Since it was difficult to determine static position of the hydrogen atoms of
the water without clear interactions, the model have no hydrogens.
;

_vrf_PLAT420_Artemether@Co-3TPHAP
;
PROBLEM:
PLAT420_ALERT_2_B D-H Bond Without Acceptor O1L --H1LB . Please Check
PLAT420_ALERT_2_B D-H Bond Without Acceptor O3L --H3LB . Please Check

RESPONSE:
The hydrogen acceptors were not observed clearly due to severely disorder.
;

_vrf_PLAT430_Artemether@Co-3TPHAP
;
PROBLEM:
PLAT430_ALERT_2_B Short Inter D...A Contact O13W ..N28A . 2.80 Ang.
-1/2+x,-1/2+y,z = 3_445 Check
PLAT430_ALERT_2_B Short Inter D...A Contact O13W ..O8W . 2.81 Ang.
x,y,z = 1_555 Check
PLAT430_ALERT_2_B Short Inter D...A Contact N20B ..O1W . 2.82 Ang.
x,y,z = 1_555 Check

RESPONSE:
These interactions should be hydrogen bonds;
however, the hydrogen of waters could not be molded due to disorder, these alerts are inevitable.

;

CCDC 2246427 (artesunate@Co-3TPHAP)

_vrf_PLAT029_Artesunate@Co-3TPHAP
;
PROBLEM:
PLAT029_ALERT_3_A _diffrn_measured_fraction_theta_full value Low . 0.933 Why?

RESPONSE:
In this measurement, the crystal was measured by omega scan.
Omega scan measurement provided the best data quality and it is suitable for this research.
However, diffractiond data set of this crystal was slightly not enough to cover full sphere.
;

_vrf_PLAT602_Artesunate@Co-3TPHAP
;
PROBLEM:
PLAT602_ALERT_2_A Solvent Accessible VOID(S) in Structure .... ! Check

RESPONSE:
We assigned solvents inside the pore as much as possible.
however, several solvents could not be modeled due to severe disorder.
;

_vrf_PLAT220_Artesunate@Co-3TPHAP
;
PROBLEM:
PLAT220_ALERT_2_B NonSolvent Resd 1 C Ueq(max)/Ueq(min) Range 6.4 Ratio

RESPONSE:
Since terminal parts of guests motioned dynamically, this alert could not be avoided.
;

_vrf_PLAT306_Artesunate@Co-3TPHAP
;
PROBLEM:
PLAT306_ALERT_2_B Isolated Oxygen Atom (H-atoms Missing ?) .... O1W Check
PLAT306_ALERT_2_B Isolated Oxygen Atom (H-atoms Missing ?) .... O5W Check
PLAT306_ALERT_2_B Isolated Oxygen Atom (H-atoms Missing ?) .... O8W Check
PLAT306_ALERT_2_B Isolated Oxygen Atom (H-atoms Missing ?) .... O9W Check
PLAT306_ALERT_2_B Isolated Oxygen Atom (H-atoms Missing ?) .... O10W Check
PLAT306_ALERT_2_B Isolated Oxygen Atom (H-atoms Missing ?) .... O11W Check
PLAT306_ALERT_2_B Isolated Oxygen Atom (H-atoms Missing ?) .... O12W Check

RESPONSE:
These oxygen atoms are components of the water in the pore of the framework.
Since it was difficult to determine static position of the hydrogen atoms of
the water without clear interactions, the model have no hydrogens.
;

_vrf_PLAT430_shelx
;
PROBLEM:
PLAT430_ALERT_2_B Short Inter D...A Contact O5S ..O10W . 2.84 Ang.
x,y,z = 1_555 Check
PLAT430_ALERT_2_B Short Inter D...A Contact O5W ..O6W . 2.74 Ang.
x,y,z = 1_555 Check
PLAT430_ALERT_2_B Short Inter D...A Contact O8W ..O9W . 2.80 Ang.
x,y,z = 1_555 Check
PLAT430_ALERT_2_B Short Inter D...A Contact O11W ..O13S . 2.84 Ang.
x,y,z = 1_555 Check
PLAT430_ALERT_2_B Short Inter D...A Contact O11W ..N20C . 2.85 Ang.
x,y,z = 1_555 Check

PLAT430_ALERT_2_B Short Inter D...A Contact O12W ..N8D . 2.87 Ang.
1-x,-1/2+y,1-z = 2_646 Check
PLAT430_ALERT_2_B Short Inter D...A Contact N30B ..O16W . 2.85 Ang.
x,y,z = 1_555 Check

RESPONSE:
These interactions should be hydrogen bonds;
however, the hydrogen of waters could not be molded due to disorder, these alerts are inevitable.
;

CCDC 2246428 (dihydroartemisinin@Co-3TPHAP)

_vrf_PLAT029_Dihydroartemisinin@Co-3TPHAP
;
PROBLEM:
PLAT029_ALERT_3_A _diffrn_measured_fraction_theta_full value Low . 0.928 Why?

RESPONSE:
In this measurement, the crystal was measured by omega scan.
Omega scan measurement provided the best data quality and it is suitable for this research.
However, diffractiond data set of this crystal was slightly not enough to cover full sphere.
;

_vrf_PLAT602_Dihydroartemisinin@Co-3TPHAP
;
PROBLEM:
PLAT602_ALERT_2_A Solvent Accessible VOID(S) in Structure .... ! Check

RESPONSE:
We assigned solvents inside the pore as much as possible.
however, several solvents could not be modeled due to severe disorder.
;

_vrf_PLAT306_Dihydroartemisinin@Co-3TPHAP
;
PROBLEM:
PLAT306_ALERT_2_B Isolated Oxygen Atom (H-atoms Missing ?) .... O3W Check
PLAT306_ALERT_2_B Isolated Oxygen Atom (H-atoms Missing ?) .... O4W Check
PLAT306_ALERT_2_B Isolated Oxygen Atom (H-atoms Missing ?) .... O5W Check
PLAT306_ALERT_2_B Isolated Oxygen Atom (H-atoms Missing ?) .... O8W Check
PLAT306_ALERT_2_B Isolated Oxygen Atom (H-atoms Missing ?) .... O10W Check
PLAT306_ALERT_2_B Isolated Oxygen Atom (H-atoms Missing ?) .... O25W Check

RESPONSE:
These oxygen atoms are components of the water in the pore of the framework.
Since it was difficult to determine static position of the hydrogen atoms of
the water without clear interactions, the model have no hydrogens.
;

_vrf_PLAT420_Dihydroartemisinin@Co-3TPHAP
;
PROBLEM:
PLAT420_ALERT_2_B D-H Bond Without Acceptor O1Y --H1Y . Please Check
PLAT420_ALERT_2_B D-H Bond Without Acceptor O1LE --H1LI . Please Check
PLAT420_ALERT_2_B D-H Bond Without Acceptor O1LF --H1LL . Please Check

RESPONSE:
The hydrogen acceptors were not observed clearly due to severely disorder.
;

_vrf_PLAT430_Dihydroartemisinin@Co-3TPHAP
;
PROBLEM:
PLAT430_ALERT_2_B Short Inter D...A Contact O2I ..O15W . 2.78 Ang.

x,y,z = 1_555 Check
PLAT430_ALERT_2_B Short Inter D...A Contact O3W ..N8B . 2.83 Ang.
1-x,-1/2+y,2-z = 2_647 Check
PLAT430_ALERT_2_B Short Inter D...A Contact O6W ..N30D . 2.85 Ang.
1-x,1/2+y,1-z = 2_656 Check
PLAT430_ALERT_2_B Short Inter D...A Contact O8W ..N8D . 2.85 Ang.
1-x,1/2+y,1-z = 2_656 Check
PLAT430_ALERT_2_B Short Inter D...A Contact O9W ..O10W . 2.66 Ang.
-x,-1/2+y,1-z = 2_546 Check
PLAT430_ALERT_2_B Short Inter D...A Contact O12W ..N30A . 2.86 Ang.
-x,1/2+y,1-z = 2_556 Check
PLAT430_ALERT_2_B Short Inter D...A Contact O20W ..N18C . 2.87 Ang.
x,y,z = 1_555 Check
PLAT430_ALERT_2_B Short Inter D...A Contact O24W ..O17W . 2.67 Ang.
-x,1/2+y,-z = 2_555 Check
PLAT430_ALERT_2_B Short Inter D...A Contact N20D ..O14W . 2.84 Ang.
x,y,z = 1_555 Check
PLAT430_ALERT_2_B Short Inter D...A Contact N24D ..O15W . 2.77 Ang.
x,y,z = 1_555 Check
PLAT430_ALERT_2_B Short Inter D...A Contact N28A ..O23W . 2.87 Ang.
-x,1/2+y,1-z = 2_556 Check
PLAT430_ALERT_2_B Short Inter D...A Contact O7W ..O17X . 2.83 Ang.
1-x,-1/2+y,1-z = 2_646 Check

RESPONSE:
These interactions should be hydrogen bonds;
however, the hydrogen of waters could not be molded due to disorder, these alerts are inevitable.
;

CCDC 2246429 (dexamethasone@Co-3TPHAP)

_vrf_PLAT602_Dexamethasone@Co-3TPHAP
;
PROBLEM:
PLAT602_ALERT_2_A Solvent Accessible VOID(S) in Structure .... ! Check

RESPONSE:
We assigned solvents inside the pore as much as possible.
however, several solvents could not be modeled due to severe disorder.
;

_vrf_PLAT029_Dexamethasone@Co-3TPHAP
;
PROBLEM:
PLAT029_ALERT_3_B _diffrn_measured_fraction_theta_full value Low . 0.943 Why?

RESPONSE:
In this measurement, the crystal was measured by omega scan.
Omega scan measurement provided the best data quality and it is suitable for this research.
However, diffractiond data set of this crystal was slightly not enough to cover full sphere.
;

_vrf_PLAT306_Dexamethasone@Co-3TPHAP
;
PROBLEM:
PLAT306_ALERT_2_B Isolated Oxygen Atom (H-atoms Missing ?) .... O1W Check
PLAT306_ALERT_2_B Isolated Oxygen Atom (H-atoms Missing ?) .... O4W Check
PLAT306_ALERT_2_B Isolated Oxygen Atom (H-atoms Missing ?) .... O8W Check

RESPONSE:
These oxygen atoms are components of the water in the pore of the framework.
Since it was difficult to determine static position of the hydrogen atoms of
the water without clear interactions, the model have no hydrogens.
;

_vrf_PLAT420_Dexamethasone@Co-3TPHAP
;
PROBLEM:
PLAT420_ALERT_2_B D-H Bond Without Acceptor O2L --H2LA . Please Check
PLAT420_ALERT_2_B D-H Bond Without Acceptor O9Z --H9Z . Please Check
PLAT420_ALERT_2_B D-H Bond Without Acceptor O14Z --H14Z . Please Check

RESPONSE:

;

_vrf_PLAT430_Dexamethasone@Co-3TPHAP
;
PROBLEM:
PLAT430_ALERT_2_B Short Inter D...A Contact O1W ..O43Q . 2.62 Ang.
1-x,-1/2+y,1-z = 2_646 Check
PLAT430_ALERT_2_B Short Inter D...A Contact O1W ..N8B . 2.85 Ang.
-x,-1/2+y,1-z = 2_546 Check
PLAT430_ALERT_2_B Short Inter D...A Contact O1Z ..O8W . 2.82 Ang.
x,y,z = 1_555 Check
PLAT430_ALERT_2_B Short Inter D...A Contact O7W ..N28C . 2.85 Ang.
x,y,z = 1_555 Check
PLAT430_ALERT_2_B Short Inter D...A Contact O9S ..O15W . 2.80 Ang.
x,y,z = 1_555 Check
PLAT430_ALERT_2_B Short Inter D...A Contact O15W ..O16W . 2.74 Ang.
x,y,z = 1_555 Check
PLAT430_ALERT_2_B Short Inter D...A Contact N20D ..O17W . 2.81 Ang.
x,y,z = 1_555 Check
PLAT430_ALERT_2_B Short Inter D...A Contact N28C ..O36Q . 2.85 Ang.
x,y,z = 1_555 Check

RESPONSE:

These interactions should be hydrogen bonds;
however, the hydrogen of waters could not be molded due to disorder, these alerts are inevitable.
;

CCDC 2246430 (betamethasone@Co-3TPHAP)

_vrf_PLAT602_Betamethasone@Co-3TPHAP
;
PROBLEM:
PLAT602_ALERT_2_A Solvent Accessible VOID(S) in Structure .... ! Check

RESPONSE:
We assigned solvents inside the pore as much as possible.
however, several solvents could not be modeled due to severe disorder.
;

_vrf_PLAT029_Betamethasone@Co-3TPHAP
;
PROBLEM:
PLAT029_ALERT_3_B _diffrn_measured_fraction_theta_full value Low . 0.942 Why?

RESPONSE:
In this measurement, the crystal was measured by omega scan.
Omega scan measurement provided the best data quality and it is suitable for this research.
However, diffractiond data set of this crystal was slightly not enough to cover full sphere.
;

_vrf_PLAT306_Betamethasone@Co-3TPHAP
;
PROBLEM:
PLAT306_ALERT_2_B Isolated Oxygen Atom (H-atoms Missing ?) .... O15W Check
PLAT306_ALERT_2_B Isolated Oxygen Atom (H-atoms Missing ?) .... O17W Check
PLAT306_ALERT_2_B Isolated Oxygen Atom (H-atoms Missing ?) .... O18W Check

PLAT306_ALERT_2_B Isolated Oxygen Atom (H-atoms Missing ?) .... O19W Check

RESPONSE:
These oxygen atoms are components of the water in the pore of the framework. Since it was difficult to determine static position of the hydrogen atoms of the water without clear interactions, the model have no hydrogens.
;

_vrf_PLAT420_Betamethasone@Co-3TPHAP
;
PROBLEM:
PLAT420_ALERT_2_B D-H Bond Without Acceptor O3L --H3LA . Please Check
PLAT420_ALERT_2_B D-H Bond Without Acceptor O3L --H3LB . Please Check
PLAT420_ALERT_2_B D-H Bond Without Acceptor O9Z --H9Z . Please Check
PLAT420_ALERT_2_B D-H Bond Without Acceptor O18Z --H18Z . Please Check

RESPONSE:
The hydrogen acceptors were not observed clearly due to severely disorder.
;

_vrf_PLAT430_Betamethasone@Co-3TPHAP
;
PROBLEM:
PLAT430_ALERT_2_B Short Inter D...A Contact O1Z ..O19W . 2.84 Ang.
1-x,-1/2+y,1-z = 2_646 Check
PLAT430_ALERT_2_B Short Inter D...A Contact O05Q ..O5S . 2.80 Ang.
x,y,z = 1_555 Check
PLAT430_ALERT_2_B Short Inter D...A Contact O3W ..O4W . 2.69 Ang.
x,y,z = 1_555 Check
PLAT430_ALERT_2_B Short Inter D...A Contact O6W ..N18D . 2.68 Ang.
x,y,z = 1_555 Check

PLAT430_ALERT_2_B Short Inter D...A Contact O13W ..N28D . 2.80 Ang.
x,y,z = 1_555 Check
PLAT430_ALERT_2_B Short Inter D...A Contact O15W ..O9S . 2.83 Ang.
1-x,-1/2+y,2-z = 2_647 Check
PLAT430_ALERT_2_B Short Inter D...A Contact O17W ..O31Q . 2.66 Ang.
1-x,1/2+y,2-z = 2_657 Check
PLAT430_ALERT_2_B Short Inter D...A Contact O17W ..O16W . 2.69 Ang.
1-x,1/2+y,2-z = 2_657 Check
PLAT430_ALERT_2_B Short Inter D...A Contact N20C ..O1W . 2.80 Ang.
x,y,z = 1_555 Check

RESPONSE:
These interactions should be hydrogen bonds;
however, the hydrogen of waters could not be molded due to disorder, these alerts are inevitable.
;

CCDC 2246431 (betamethasone dipropionate@Co-3TPHAP)

_vrf_PLAT602_Betamethasone_17,21-dipropionate@Co-3TPHAP
;
PROBLEM:
PLAT602_ALERT_2_A Solvent Accessible VOID(S) in Structure .... ! Check

RESPONSE:
We assigned solvents inside the pore as much as possible.
however, several solvents could not be modeled due to severe disorder.
;

_vrf_PLAT306_Betamethasone_17,21-dipropionate@Co-3TPHAP
;
PROBLEM:
PLAT306_ALERT_2_B Isolated Oxygen Atom (H-atoms Missing ?) .... O3W Check
PLAT306_ALERT_2_B Isolated Oxygen Atom (H-atoms Missing ?) .... O6W Check

RESPONSE:
These oxygen atoms are components of the water in the pore of the framework.
Since it was difficult to determine static position of the hydrogen atoms of
the water without clear interactions, the model have no hydrogens
;

_vrf_PLAT430_Betamethasone_17,21-dipropionate@Co-3TPHAP
;
PROBLEM:
PLAT430_ALERT_2_B Short Inter D...A Contact O2C ..O2L . 2.81 Ang.
x,y,z = 1_555 Check
PLAT430_ALERT_2_B Short Inter D...A Contact O2L ..O1W . 2.63 Ang.
x,y,z = 1_555 Check
PLAT430_ALERT_2_B Short Inter D...A Contact O2L ..O5W . 2.82 Ang.
1-x,1+y,1-z = 2_666 Check
PLAT430_ALERT_2_B Short Inter D...A Contact O2L ..O4W . 2.84 Ang.
x,y,z = 1_555 Check
PLAT430_ALERT_2_B Short Inter D...A Contact O6W ..O20Z . 2.80 Ang.
x,-1+y,z = 1_545 Check
PLAT430_ALERT_2_B Short Inter D...A Contact O6W ..N8B . 2.83 Ang.
-1/2+x,-1/2+y,z = 3_445 Check

RESPONSE:
These interactions should be hydrogen bonds;
however, the hydrogen of waters could not be molded due to disorder, these alerts are inevitable.
;

CCDC 2246432 (spironolactone@Co-3TPHAP)

_vrf_PLAT602_Spironolactone@Co-3TPHAP
;
PROBLEM:
PLAT602_ALERT_2_A Solvent Accessible VOID(S) in Structure .... ! Check

RESPONSE:
We assigned solvents inside the pore as much as possible.
however, several solvents could not be modeled due to severe disorder.
;

_vrf_PLAT306_Spironolactone@Co-3TPHAP
;
PROBLEM:
PLAT306_ALERT_2_B Isolated Oxygen Atom (H-atoms Missing ?) .... O1W Check
PLAT306_ALERT_2_B Isolated Oxygen Atom (H-atoms Missing ?) .... O2W Check
PLAT306_ALERT_2_B Isolated Oxygen Atom (H-atoms Missing ?) .... O9W Check

RESPONSE:
These oxygen atoms are components of the water in the pore of the framework.
Since it was difficult to determine static position of the hydrogen atoms of
the water without clear interactions, the model have no hydrogens.
;

_vrf_PLAT420_Spironolactone@Co-3TPHAP
;
PROBLEM:
PLAT420_ALERT_2_B D-H Bond Without Acceptor O11L --H11B . Please Check

RESPONSE:
The hydrogen acceptors were not observed clearly due to severely disorder.
;

_vrf_PLAT430_Spironolactone@Co-3TPHAP
;
PROBLEM:

PLAT430_ALERT_2_B Short Inter D...A Contact N18B ..O59Q . 2.75 Ang.
x,y,z = 1_555 Check

RESPONSE:
These interactions should be hydrogen bonds;
however, the hydrogen of waters could not be molded due to disorder, these alerts are inevitable.
;

CCDC 2246433 ((+)-griseofulvin@Co-3TPHAP)

_vrf_PLAT602_+-Griseofulvin@Co-3TPHAP
;
PROBLEM:
PLAT602_ALERT_2_A Solvent Accessible VOID(S) in Structure .... ! Check

RESPONSE:
We assigned solvents inside the pore as much as possible.
however, several solvents could not be modeled due to severe disorder.
;

_vrf_PLAT306_+-Griseofulvin@Co-3TPHAP
;
PROBLEM:
PLAT306_ALERT_2_B Isolated Oxygen Atom (H-atoms Missing ?) .... O3W Check
PLAT306_ALERT_2_B Isolated Oxygen Atom (H-atoms Missing ?) .... O6W Check

RESPONSE:
These oxygen atoms are components of the water in the pore of the framework.
Since it was difficult to determine static position of the hydrogen atoms of
the water without clear interactions, the model have no hydrogens.
;

_vrf_PLAT430_+-Griseofulvin@Co-3TPHAP

;
PROBLEM:
PLAT430_ALERT_2_B Short Inter D...A Contact O2C ..O2L . 2.81 Ang.
x,y,z = 1_555 Check
PLAT430_ALERT_2_B Short Inter D...A Contact O2L ..O1W . 2.63 Ang.
x,y,z = 1_555 Check
PLAT430_ALERT_2_B Short Inter D...A Contact O2L ..O5W . 2.82 Ang.
1-x,1+y,1-z = 2_666 Check
PLAT430_ALERT_2_B Short Inter D...A Contact O2L ..O4W . 2.84 Ang.
x,y,z = 1_555 Check
PLAT430_ALERT_2_B Short Inter D...A Contact O6W ..O20Z . 2.80 Ang.
x,-1+y,z = 1_545 Check
PLAT430_ALERT_2_B Short Inter D...A Contact O6W ..N8B . 2.83 Ang.
-1/2+x,-1/2+y,z = 3_445 Check

RESPONSE:
These interactions should be hydrogen bonds;
however, the hydrogen of waters could not be molded due to disorder, these alerts are inevitable.
;

CCDC 2246434 (celecoxib@Co-3TPHAP)

_vrf_PLAT306_Celecoxib@Co-3TPHAP
;
PROBLEM:
PLAT306_ALERT_2_B Isolated Oxygen Atom (H-atoms Missing ?) .... O1W Check
PLAT306_ALERT_2_B Isolated Oxygen Atom (H-atoms Missing ?) .... O2W Check
PLAT306_ALERT_2_B Isolated Oxygen Atom (H-atoms Missing ?) .... O7W Check

RESPONSE:
These oxygen atoms are components of the water in the pore of the framework.
Since it was difficult to determine static position of the hydrogen atoms of
the water without clear interactions, the model have no hydrogens.
;

_vrf_PLAT430_Celecoxib@Co-3TPHAP
;
PROBLEM:
PLAT430_ALERT_2_B Short Inter D...A Contact O1S ..O1W . 2.77 Ang.
1-x,-1/2+y,1/2-z = 2_645 Check
PLAT430_ALERT_2_B Short Inter D...A Contact O1W ..O4W . 2.77 Ang.
x,y,z = 1_555 Check
PLAT430_ALERT_2_B Short Inter D...A Contact O1W ..N18A . 2.85 Ang.
x,y,z = 1_555 Check
PLAT430_ALERT_2_B Short Inter D...A Contact O2D ..O9Q . 2.76 Ang.
x,y,z = 1_555 Check
PLAT430_ALERT_2_B Short Inter D...A Contact O7W ..O26Z . 2.69 Ang.
x,1+y,z = 1_565 Check
PLAT430_ALERT_2_B Short Inter D...A Contact O9W ..N30A . 2.84 Ang.
1-x,1-y,1-z = 3_666 Check
PLAT430_ALERT_2_B Short Inter D...A Contact N28B ..O12Q . 275 Ang.
2-x,1-y,1-z = 3_766 Check
PLAT430_ALERT_2_B Short Inter D...A Contact N30B ..O12Q . 2.69 Ang.
2-x,1-y,1-z = 3_766 Check

RESPONSE:
These interactions should be hydrogen bonds;
however, the hydrogen of waters could not be molded due to disorder,
these alerts are inevitable.
;

CCDC 2246435 (meloxicam@Co-3TPHAP)

_vrf_PLAT602_Meloxicam@Co-3TPHAP
;
PROBLEM:
PLAT602_ALERT_2_A Solvent Accessible VOID(S) in Structure .... ! Check

RESPONSE:

We assigned solvents inside the pore as much as possible.
however, several solvents could not be modeled due to severe disorder.
;

_vrf_PLAT306_Meloxicam@Co-3TPHAP
;
PROBLEM:
PLAT306_ALERT_2_B Isolated Oxygen Atom (H-atoms Missing ?) .... O10W Check

RESPONSE:
These oxygen atoms are components of the water in the pore of the framework.
Since it was difficult to determine static position of the hydrogen atoms of
the water without clear interactions, the model have no hydrogens.
;

_vrf_PLAT430_Meloxicam@Co-3TPHAP
;
PROBLEM:
PLAT430_ALERT_2_B Short Inter D...A Contact O10W ..N8A . 2.82 Ang.
1-x,-1/2+y,3/2-z = 2_646 Check
PLAT430_ALERT_2_B Short Inter D...A Contact N20A ..O7W . 2.83 Ang.
x,y,z = 1_555 Check
PLAT430_ALERT_2_B Short Inter D...A Contact N30B ..O1S . 2.73 Ang.
-x,1-y,1-z = 3_566 Check

RESPONSE:
These interactions should be hydrogen bonds;
however, the hydrogen of waters could not be molded due to disorder, these alerts are inevitable.
;

CCDC 2246417 (hemiactinorhodin methyl ester@Co-3TPHAP)

_vrf_PLAT029_Hemi-actinorhodin_methyl_ester@Co-3TPHAP

;
PROBLEM:
PLAT029_ALERT_3_A _diffrn_measured_fraction_theta_full value Low . 0.935 Why?

RESPONSE:
In this measurement, the crystal was measured by omega scan.
Omega scan measurement provided the best data quality and it is suitable for this research.
However, diffractiond data set of this crystal was slightly not enough to cover full sphere.
;

_vrf_PLAT035_Hemi-actinorhodin_methyl_ester@Co-3TPHAP
;
PROBLEM:
PLAT035_ALERT_1_B _chemical_absolute_configuration Info Not Given Please Do !

RESPONSE:
This crystal data is an encapsulation of enantiomers.
To certify the chiral recognition ability of Co-3TPHAP,
the model was analyzed as a chiral space group to give Flack parameter.
As a result, Flack parameter suggests the enantiomers were included equally,
which supports the chiral recognition theory of this MOF.
;

_vrf_PLAT221_Hemi-actinorhodin_methyl_ester@Co-3TPHAP
;
PROBLEM:
PLAT221_ALERT_2_B Solv./Anion Resd 5 C Ueq(max)/Ueq(min) Range 10.0 Ratio

RESPONSE:
Some of the low occupancy guest models were affected by solvent electron density or systematic error due to the low completeness,
thus this alert could not be avoidable.

Since the high occupancy model is reasonable enough, this result does not affect the claim in this manuscript and model validity.
;

_vrf_PLAT306_Hemi-actinorhodin_methyl_ester@Co-3TPHAP
;
PROBLEM:
PLAT306_ALERT_2_B Isolated Oxygen Atom (H-atoms Missing ?) .... O3W Check
PLAT306_ALERT_2_B Isolated Oxygen Atom (H-atoms Missing ?) .... O4W Check
PLAT306_ALERT_2_B Isolated Oxygen Atom (H-atoms Missing ?) .... O6W Check
PLAT306_ALERT_2_B Isolated Oxygen Atom (H-atoms Missing ?) .... O7W Check

RESPONSE:
These oxygen atoms are components of the water in the pore of the framework.
Since it was difficult to determine static position of the hydrogen atoms of
the water without clear interactions, the model have no hydrogens.
;

_vrf_PLAT430_Hemi-actinorhodin_methyl_ester@Co-3TPHAP
;
PROBLEM:
PLAT430_ALERT_2_B Short Inter D...A Contact O3W ..N30C . 2.84 Ang.
x,y,z = 1_555 Check
PLAT430_ALERT_2_B Short Inter D...A Contact O4W ..O8Q . 2.81 Ang.
1-x,1/2+y,2-z = 2_657 Check
PLAT430_ALERT_2_B Short Inter D...A Contact O6W ..O5W . 2.59 Ang.
x,y,z = 1_555 Check
PLAT430_ALERT_2_B Short Inter D...A Contact O6W ..O1U . 2.81 Ang.
-x,1/2+y,1-z = 2_556 Check
PLAT430_ALERT_2_B Short Inter D...A Contact O7W ..N30A . 2.85 Ang.
-x,1/2+y,1-z = 2_556 Check
PLAT430_ALERT_2_B Short Inter D...A Contact O10Q ..N8B . 2.85 Ang.
x,y,1+z = 1_556 Check
PLAT430_ALERT_2_B Short Inter D...A Contact O41Q ..N3E . 2.83 Ang.

1-x,1/2+y,1-z = 2_656 Check
PLAT430_ALERT_2_B Short Inter D...A Contact N3E ..O42Q . 2.86 Ang.
1-x,-1/2+y,1-z = 2_646 Check
PLAT430_ALERT_2_B Short Inter D...A Contact N8B ..O11Q . 2.86 Ang.
x,y,-1+z = 1_554 Check
PLAT430_ALERT_2_B Short Inter D...A Contact N18A ..O1W . 2.79 Ang.
-x,1/2+y,1-z = 2_556 Check

RESPONSE:
These interactions should be hydrogen bonds;
however, the hydrogen of waters could not be molded due to disorder,
these alerts are inevitable.
;
_vrf_PLAT601_Hemi-actinorhodin_methyl_ester@Co-3TPHAP
;
PROBLEM:
PLAT601_ALERT_2_B Unit Cell Contains Solvent Accessible VOIDS of . 169 Ang**3

RESPONSE:
We assigned solvents inside the pore as much as possible.
however, several solvents could not be modeled due to severe disorder.
;
_vrf_PLAT213_Hemi-actinorhodin_methyl_ester@Co-3TPHAP
;
PROBLEM:
PLAT213_ALERT_2_B Atom C11M has ADP max/min Ratio ..... 4.2 prolat
PLAT213_ALERT_2_B Atom C11C has ADP max/min Ratio ..... 4.2 prolat

RESPONSE:
These atoms are the 3-pyridyl groups of the ligand,
and this alert is come from the rotation of the functional group.
;

<u>CCDC 2246418 (hemi-actinorhodin@Co-3TPHAP)</u>

_vrf_PLAT035_Hemi-actinorhodin@Co-3TPHAP
;
PROBLEM:
PLAT035_ALERT_1_B _chemical_absolute_configuration Info Not Given Please Do !

RESPONSE:
This crystal data is an encapsulation of enantiomers.
To certify the chiral recognition ability of Co-3TPHAP,
the model was analyzed as a chiral space group to give Flack parameter.
As a result, Flack parameter suggests the enantiomers were included equally,
which supports the chiral recognition theory of this MOF.
;

_vrf_PLAT306_Hemi-actinorhodin@Co-3TPHAP
;
PROBLEM:
PLAT306_ALERT_2_B Isolated Oxygen Atom (H-atoms Missing ?) .... O2W Check
PLAT306_ALERT_2_B Isolated Oxygen Atom (H-atoms Missing ?) .... O3W Check

RESPONSE:
These oxygen atoms are components of the water in the pore of the framework.
Since it was difficult to determine static position of the hydrogen atoms of
the water without clear interactions, the model have no hydrogens.
;

_vrf_PLAT341_Hemi-actinorhodin@Co-3TPHAP
;
PROBLEM:
PLAT341_ALERT_3_B Low Bond Precision on C-C Bonds .... 0.01588 Ang.

RESPONSE:

Some parts of the MOF were disconnected by ligand exchange.
As a result, the robustness of the MOF got lower and the entire framework was subtly distorted,
thus this alert was not avoided.
;

_vrf_PLAT430_Hemi-actinorhodin@Co-3TPHAP
;
PROBLEM:
PLAT430_ALERT_2_B Short Inter D...A Contact O2W ..N8B . 2.85 Ang.
1/2+x,1/2+y,z = 3_555 Check
PLAT430_ALERT_2_B Short Inter D...A Contact O32Q ..N20A . 2.76 Ang.
x,y,z = 1_555 Check
PLAT430_ALERT_2_B Short Inter D...A Contact N20B ..O41Q . 2.85 Ang.
x,y,z = 1_555 Check
PLAT430_ALERT_2_B Short Inter D...A Contact N30A ..O52Q . 2.85 Ang.
1/2+x,-1/2+y,z = 3_545 Check

RESPONSE:
These interactions should be hydrogen bonds;
however, the hydrogen of waters could not be molded due to disorder,
these alerts are inevitable.
;

CCDC 2246419 (neopeltolide macrolactone@Co-3TPHAP)

_vrf_PLAT029_Neopeltolide_macrolactone@Co-3TPHAP
;
PROBLEM:
PLAT029_ALERT_3_A _diffrn_measured_fraction_theta_full value Low . 0.931 Why?

RESPONSE:
In this measurement, the crystal was measured by omega scan.
Omega scan measurement provided the best data quality and it is suitable for this research.
However, diffractiond data set of this crystal was slightly not enough to cover full sphere.

;

_vrf_PLAT602_Neopeltolide_macrolactone@Co-3TPHAP
;
PROBLEM:
PLAT602_ALERT_2_A Solvent Accessible VOID(S) in Structure .... ! Check

RESPONSE:
We assigned solvents inside the pore as much as possible.
however, several solvents could not be modeled due to severe disorder.
;

_vrf_PLAT213_Neopeltolide_macrolactone@Co-3TPHAP
;
PROBLEM:
Atom C6Y has ADP max/min Ratio ..... 4.3 prolat

RESPONSE:
Since terminal parts of guests motioned dynamically, this alert could not be avoided.
;

_vrf_PLAT220_Neopeltolide_macrolactone@Co-3TPHAP
;
PROBLEM:
PLAT213_ALERT_2_B Atom C6Y has ADP max/min Ratio ..... 4.3 prolat

RESPONSE:
Since terminal parts of guests motioned dynamically, this alert could not be avoided.
;

_vrf_PLAT420_Neopeltolide_macrolactone@Co-3TPHAP
;
PROBLEM:
PLAT420_ALERT_2_B D-H Bond Without Acceptor O6L --H6LB . Please Check
PLAT420_ALERT_2_B D-H Bond Without Acceptor O7L --H7LB . Please Check
PLAT420_ALERT_2_B D-H Bond Without Acceptor O8L --H8LA . Please Check

RESPONSE:
The hydrogen acceptors were not observed clearly due to severely disorder.
;

_vrf_PLAT430_Neopeltolide_macrolactone@Co-3TPHAP
;
PROBLEM:
PLAT430_ALERT_2_B Short Inter D...A Contact O1L ..O51Q . 2.68 Ang.
x,y,z = 1_555 Check
PLAT430_ALERT_2_B Short Inter D...A Contact O7W ..N8B . 2.85 Ang.
-1+x,y,-1+z = 1_454 Check
PLAT430_ALERT_2_B Short Inter D...A Contact N8D ..O1X . 2.80 Ang.
1-x,-1/2+y,1-z = 2_646 Check
PLAT430_ALERT_2_B Short Inter D...A Contact N18A ..O48Q . 2.74 Ang.
x,y,z = 1_555 Check
PLAT430_ALERT_2_B Short Inter D...A Contact N28C ..O1V . 2.58 Ang.
-x,-1/2+y,1-z = 2_546 Check

RESPONSE:
These interactions should be hydrogen bonds;
however, the hydrogen of waters could not be molded due to disorder, these alerts are inevitable.
;

CCDC 2246420 (artemisinin)

No alerts were reported.

CCDC 2246421 (dexamethasone)

No alerts were reported.

CCDC 2246422 (betamethasone dipropionate)

No alerts were reported.

CCDC 2246423 (meloxicam)

_vrf_PLAT029_Meloxicam
;
PROBLEM:
PLAT029_ALERT_3_A _diffrn_measured_fraction_theta_full value Low . 0.918 Why?

RESPONSE:
In this measurement, the crystal was measured by omega scan.
Therefore, diffractiond data set of this crystal was slightly not enough to cover full sphere.
;

_audit_creation_date 2022-11-10
_audit_creation_method
;

CCDC 2246424 ((+)-griseofulvin)

_vrf_PLAT029_+-Griseofulvin
;
PROBLEM:
PLAT029_ALERT_3_B _diffrn_measured_fraction_theta_full value Low . 0.946 Why?

RESPONSE:
In this measurement, the crystal was measured by omega scan.
Therefore, diffractiond data set of this crystal was slightly not enough to cover full sphere.
;

Supplementary References

1. Wada, Y. *et al.* Multi-interactive Coordination Network Featuring a Ligand with Topologically Isolated p Orbitals. *Inorg. Chem.* **60**, 17858–17864 (2021).
